# Multilocus sequence typing of the invasive pest *Halyomorpha halys* (Hemiptera: Pentatomidae) and associated endosymbiont reveals unexplored diversity

**DOI:** 10.1111/1744-7917.70034

**Published:** 2025-04-09

**Authors:** Matteo Dho, Matteo Montagna, Chenxi Liu, Giulia Magoga, Giobbe Forni, Alberto Alma, Elena Gonella

**Affiliations:** ^1^ Department of Agricultural, Forest and Food Sciences University of Torino Torino Italy; ^2^ Department of Agricultural Sciences University of Naples Federico II Portici Italy; ^3^ USDA‐ARS Sino‐American Biological Control Laboratory, Institute of Plant Protection Chinese Academy of Agricultural Sciences Beijing China; ^4^ Department of Biological, Geological and Environmental Sciences University of Bologna Italy

**Keywords:** brown marmorated stink bug, *Candidatus* Pantoea carbekii, COI, nuclear markers, population genetic diversity

## Abstract

*Halyomorpha halys* is an invasive pest affecting a wide range of crops in many regions of the world. Rapid and cost‐effective methods to reconstruct its invasion routes are crucial for implementing strategies to prevent further spread. The mitochondrial markers COI and COII and the pseudogene ΔybgF of the primary symbiont “*Candidatus* Pantoea carbekii” have been analyzed to track the spread of *H. halys*. However, these markers do not provide sufficient resolution to fully elucidate invasion routes. Here, *H. halys* individuals from native and invasive populations were analyzed to identify new DNA markers and evaluate their effectiveness in a multilocus sequence typing (MLST) framework. Three new nuclear markers for *H. halys* (Hh_KsPi, Hh_UP1, Hh_D3PDh) and three new markers for *P. carbekii* (Pc_TamA, Pc_SucA, Pc_SurA) were identified. Hh_D3PDh was the most informative marker for *H. halys*, describing two more haplotypes than COI. By integrating Hh_D3PDh with mitochondrial markers, 30 distinct haplotypes were identified, with each of the populations studied exhibiting multiple haplotypes. Pc_SucA was the most informative symbiont marker, and when all *P. carbekii* markers were combined, symbiont diversity was greatly increased. The low network specialization between the novel nuclear markers and both mitochondrial and symbiont markers underlined the higher power of nuclear markers. Interestingly, perfect network specialization between *H. halys* COI and symbiont markers was found in populations from invaded areas, suggesting that some holobiont variants may contribute to enhanced invasive ability. A MLST workflow is proposed as a new tool for population genetics analysis and reconstruction of *H. halys* invasion.

## Graphical abstract

A multilocus sequence typing approach is proposed to explore *Halymorpha halys* genetic diversity, taking into consideration both insect nuclear markers and markers from the gut symbiont “*Candidatus* Pantoa carbekii.” Increased information was revealed regarding the number of distinct holobiont haplotypes in native and invasive populations of the pest.

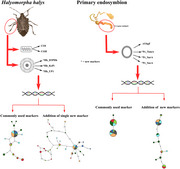

## Introduction

*Halyomorpha halys* (Stål) (Hemiptera: Pentatomidae) commonly known as the brown marmorated stink bug, is a highly polyphagous and invasive phytophagous insect native to Eastern Asia (Hoebeke & Carter, 2003; Lee *et al.*, 2013; Leskey & Nielsen, 2018). This species is harmful to a wide range of agricultural crops, such as apples, pears, peaches, tomatoes, olives and hazelnuts (Leskey *et al.*, 2012; Bariselli *et al.*, 2016; Bosco *et al.*, 2018; Damos *et al.*, 2019). In addition to its agricultural impact, *H. halys* becomes a domestic nuisance during its mass dispersal from fields to buildings for overwintering (Hancock *et al.*, 2019). First detected outside of the native range in 1996 in Pennsylvania (Hoebeke & Carter, 2003), *H. halys* is now widespread across North America (Gariepy *et al.*, 2014; Rice *et al.*, 2014; Abram *et al.*, 2017; Morey *et al.*, 2022). In Europe, it was firstly spotted in 2004 in Liechtenstein (Arnold, 2009) and in 2007 in Zurich region (Wermelinger *et al.*, 2007). The expansion rapidly increased over the years, with initial descriptions in Germany (Heckmann, 2012), Greece (Milonas & Partsinevelos, 2014), Italy (Maistrello *et al.*, 2014) and France (Callot & Brua, 2013), up to most recent detections outside European mainland in Madeira and Canary archipelagos (Petrovan *et al.*, 2022; Gaspar *et al.*, 2023). A comprehensive summary of first detections in European countries can be found in Gariepy *et al.* (2021). Recently, *H. halys* was detected in Western and Central Asian countries such as Armenia (Kalashian *et al.*, 2022), Kazakhstan (Temreshev *et al.*, 2018) and Uzbekistan (Gandjaeva *et al.*, 2022) but also in North Africa (Nouere *et al.*, 2020; van der Heyden *et al.*, 2021). In the Southern Hemisphere, *H. halys* has been recorded in Chile in 2017 (Faúndez & Rider, 2017) and adventive individuals were intercepted also in Australia and New Zealand (Horwood *et al.*, 2019).

*Halyomorpha halys* hosts its primary endosymbiont, “*Candidatus* Pantoea carbekii,” hereafter referred to as *P. carbekii*, in the caeca of the V4 midgut region (Bansal *et al.*, 2014). The genome of *P. carbekii* revealed functional pathways for synthesizing essential amino acids and vitamins that are absent from the diet, which may explain *H. halys* broad host plants range (Kenyon *et al.*, 2015). *P. carbekii* can be considered a primary endosymbiont since deprived insects show significantly decreased survival and fitness; interruption of symbiont infection is currently used as a control strategy (Gonella & Alma, 2023; Dho *et al.*, 2025).

Reconstructing the invasion patterns of *H. halys* is crucial for identifying pathways of introduction and preventing future invasions (Xu *et al.*, 2014; Gariepy *et al.*, 2015; Cesari *et al.*, 2018). For this purpose, molecular markers have been proven to be powerful tools for assessing genetic diversity among populations, facilitating the tracking of invasions (Ficetola *et al.*, 2008; Xu *et al.*, 2014). For *H. halys*, to this aim many studies have focused on amplifying and sequencing a fragment of the Cytochrome c oxidase subunit I (COI) mitochondrial region (Gariepy *et al.*, 2014; Xu *et al.*, 2014; Gariepy *et al.*, 2015; Cesari *et al.*, 2018; Lee *et al.*, 2018; Horwood *et al.*, 2019; Kapantaidaki *et al.*, 2019; Martinez‐Sañudo *et al.*, 2020; Schuler *et al.*, 2021; Yan *et al.*, 2021a; Gaspar *et al.*, 2023). The COI marker is advantageous due to the extensive availability of sequences in public databases, allowing for the construction of population‐specific profiles (Gariepy *et al.*, 2021). However, with both primary and secondary invasions occurring rapidly in new areas (Abram *et al.*, 2017; Cesari *et al.*, 2018; Schuler *et al.*, 2021) relying solely on a single marker, such as COI, has become insufficient to trace the origin of invasive populations (Horwood *et al.*, 2019; Gariepy *et al.*, 2021; Yan *et al.*, 2021a). The use of few markers can be insufficient to clearly reconstruct the population genetics, especially for species with high genetic variability (Medina *et al.*, 2006). More in depth analysis based on NGS techniques are possible for biological invasion studies (Rius *et al.*, 2015) and ddRAD sequencing has already been used for population genetics studies in *H. halys* (Yan *et al.*, 2021b; Parvizi *et al.*, 2023). On the other hand, the selection of few highly informative markers can be a faster, cost‐effective alternative that does not demand great computational resources (Pistone *et al.*, 2016), all characteristics that fit well for developing a fast and widely applicable tool.

Additional molecular markers to complement COI have been sought, with a fragment of Cytochrome c oxidase subunit II (COII) being one of the most commonly used (Cesari *et al.*, 2018; Yan *et al.*, 2021b; Berteloot *et al.*, 2024). Among nuclear markers, Internal Transcribed Spacer 1 (ITS1) has been employed in few studies (Zhu *et al.*, 2016; Kapantaidaki *et al.*, 2019). In addition, molecular markers derived from the primary symbiont have been identified and successfully used to pinpoint the source population of new invasions (Otero‐Bravo & Sabree, 2018; Laterza *et al.*, 2023). The pseudogene ΔybgF of *P. carbekii* has emerged as the best marker so far for reconstructing *H. halys* invasions through symbiont genetic variability analysis, though it has not proven as effective as the host's COI (Otero‐Bravo & Sabree, 2018), with only seven distinct haplotypes identified (Martinez‐Sañudo *et al.*, 2020). Nevertheless, by considering the primary symbiont marker not as a proxy for the host genetic variability, but as an additional layer of information, coupling ΔybgF with host COI increases the resolution of host–symbiont haplotypes and improves the ability to distinguish invasive population origins (Martinez‐Sañudo *et al.*, 2020; Laterza *et al.*, 2023).

Despite significant efforts, the current markers are insufficient, and the need for novel, more informative markers has become increasingly apparent to better defining *H. halys* invasion patterns (Valentin *et al.*, 2017; Cesari *et al.*, 2018; Laterza *et al.*, 2023). The aim of this study is to develop a cost‐effective MLST approach, suitable for population genetics analyses and invasion pattern reconstruction by identifying novel molecular markers, focusing on *H. halys* nuclear and *P. carbekii* DNA, to improve current haplotyping procedures.

## Materials and methods

### Insect collection

Seven distinct sampling sites were selected for the collection of adult *H. halys* specimens, five from invaded areas (three in Italy and two in Türkiye) and two from the insect native range (China) (Table 1). The Beijing (CN) population came from a laboratory reared colony, maintained for over 35 generations in controlled conditions. A total of 72 *H. halys* adults were used in this study. Adults were collected using the plant beating technique, transported alive to the laboratory in 25 cm × 15 cm × 13 cm plastic boxes, killed at −20 °C, and stored in pure ethanol at −20 °C until DNA extraction.

**Table 1:** *H. halys* populations size and sampling sites

Country	Province	Collection site coordinates	Number of samples
Italy	Cuneo	44°40′53.9″N, 7°51′06.6″E	10
	Cremona	45°15′12.9″N, 10°05′20.4″E	10
	Reggio Emilia	44°37′02.0″N, 10°42′16.6″E	11
Türkiye	Samsun	41°10′31.8″N, 36°30′39.8″E	8
	Ordu	40°59′54.9″N, 37°33′59.6″E	13
China	Henan	34°24′27.6″N, 110°54′45.6″E	10
	Beijing^†^	40°07′44.4″N, 116°09′50.0″E	10

^†^ Laboratory reared population, originated from adults collected in Sanming City (Fujian Province, China) (26°15′40.0″N, 117°38′37.3″E).

### Halyomorpha halys genetic analysis and nuclear marker identification

DNA was extracted from the legs of *H. halys* adults with DNeasy^®^ Blood and Tissue kit (Qiagen, Hilden, Germany), following manufacturer's instructions. The extracted DNA was used as a template for the amplification of selected mitochondrial genes, COI and COII, and newly identified nuclear markers. All the individuals were tested for each marker under analysis. Mitochondrial genes included COI and COII. For COI, new *H. halys*‐specific primers were designed using Primer BLAST function of the NCBI Genome Data Viewer. The target region ranged from position 1366 to 2139 of the reference mitochondrial region (GenBank Acc. No. NC_013272). Details of primer sequences and annealing temperature are indicated in Table S1. Thermal conditions were as follows: 34 cycles of: 95 °C for 30 s, annealing for 30 s and 72 °C for 1 min, followed by 10 min at 72 °C for final extension. For COII, the chosen primer pair and the thermal conditions were those described in Cesari *et al.* (2018).

To identify possible nuclear markers, all 20 available Hemiptera genomes with annotated CDS were downloaded from NCBI (last check 05/27/2022). All the CDS were translated and orthologous genes were inferred with Orthofinder (Emms & Kelly, 2019). Orthogroups found in *H. halys* and in at least six other species were selected. Chosen orthogroups were aligned as proteins with MUSCLE algorithm and back‐translated into nucleotides using pal2nal (Suyama *et al.*, 2006). Orthogroups were ranked according to the substitution rates with the corresponding *H. halys* gene. In total, 20 genes were chosen as possible fast‐evolving molecular markers. For each gene, primer pairs were designed on two exons flanking an intron, to increase the chance of finding variable sites. Primers were designed using Primer BLAST function of the NCBI Genome Data Viewer. Optimal thermal conditions for each primer pair were selected via gradient PCR and each primer couple was tested on 10 *H. halys* individuals having at least 2 different COI haplotypes. The three best‐performing markers in terms of variability and quality of the sequences were selected. Chosen markers were named Hh_KsPi, Hh_D3PDh and Hh_UP1. Details of primer sequences, target gene, position in the reference genome, and annealing temperature for each marker are indicated in Table S1. Thermal conditions for each nuclear marker were the same as described for novel COI, except for annealing temperature.

Amplicon size was visualized via electrophoresis with 1% agarose gel stained with GelRed^®^ (MilliporeSigma, Temecula, CA). PCR products were purified with QIAquick PCR purification kit^®^ (Qiagen) and Sanger sequenced by an external company (Eurofins Genomics, Köln, Germany).

### “Candidatus Pantoea carbekii” markers identification

To isolate DNA from the primary endosymbiont of *H. halys*, the V4 ventricle region of adult insect midgut was dissected in a Petri dish with 500 *µ*L of a sterile NaCl solution 0.9%, under a dissection microscope using fine forceps. DNA was extracted from the V4 ventricles using the DNeasy^®^ Blood and Tissue kit (Qiagen) for all *H. halys* adults under analysis. High quality DNA was obtained for 66 out of 72 individuals. All these 66 individuals were used for subsequent molecular analyses for all molecular markers. The ΔybgF marker was amplified using previously described primers and PCR conditions (Otero‐Bravo & Sabree, 2018). To identify novel markers, *P. carbekii* annotated genome was downloaded from GenBank (Acc. No. NZ_CP010907) and each coding sequence (CDS) was blasted on the other available *P. carbekii* genome (Acc. No. AP012554). All genes having at least three SNPs and/or one indel with their homolog on the second genome were kept for further analyses. From these, ten genes with the highest number of SNPs or indels within an 800 bp range were chosen. Primers for amplifying portions of these target genes were designed using Primer BLAST function of the NCBI Genome Data Viewer. Optimal thermal conditions for each primer pair were determined using gradient PCR, and each primer pair was tested on 10 *P. carbekii* individuals having at least 2 different ΔybgF haplotypes. The three best performing markers, based on sequence variability and quality, were selected. Chosen markers were named Pc_SucA, Pc_SurA, Pc_TamA. Details of primer sequences, target gene name, position in reference genome and annealing temperature for each marker are provided in Table S1. The thermal cycling conditions for amplification of each target were 34 cycles of: 95 °C for 30 s, annealing phase for 30 s and 72 °C for 1 min, followed by 10 min at 72 °C. Amplicons size visualization and sequencing were performed as described above.

### Haplotype identification and statistical analyses

All electropherograms were checked for low quality bases and flanking regions were manually trimmed before generating consensus sequences. Only for *H. halys* nuclear markers, in case electropherograms presented evidence of possible heterozygosis, such as double peaks in a specific point or consecutive double peaks starting after high quality bases, consensus sequences were generated after allele inference with the web application Indigo of Tracy (Rausch *et al.*, 2020). To limit the risk of artifact generation, electropherograms with clear inconsistences in electrophoretic mobility were excluded from the analysis. Each singleton generated by Indigo from heterozygous samples presenting indels or a combination of indels and SNPs was excluded from the analysis to reduce potential artifacts. Each sequence presenting indels and/or SNPs was manually curated by checking the original electropherogram before keeping it for the analysis. All singletons from sequences containing only SNPs were carefully checked.

Sequence translation of novel *P. carbekii* haplotypes was performed with ExPASy (Artimo *et al.*, 2012). To avoid frameshift errors during translation, any missing 5’ and 3’ regions of novel haplotypes were replaced with the corresponding regions from the reference genome. Structural damage in resulting protein structures due to amino acids missense between haplotypes was predicted using Missense3D (Ittisoponpisan *et al.*, 2019).

Newly identified marker sequences were compared with reference genomes using NCBI BLAST (*H. halys*: GCF_000696795; *P. carbekii*: NZ_CP010907). Orthologous sequences were aligned with sequences retrieved from literature using MUSCLE algorithm implemented in MEGA version 11 (Tamura *et al.*, 2021). COI and COII reference databases were created based on datasets published by Cesari *et al.* (2018), supplemented with sequences from additional studies (Kapantaidaki *et al.*, 2019; Martinez‐Sañudo *et al.*, 2020; Schuler *et al.*, 2021; Yan *et al.*, 2021a) (Tables S2 and S3). In cases where different haplotype names had been assigned to the same sequence, the nomenclature from Cesari *et al.* (2018) was used. Haplotype identification was performed with DnaSP 6 (Rozas *et al.*, 2017). To check for the presence of haplotypes described by Martinez‐Sañudo *et al.* (2020), the COI haplotype inference was preliminary performed on a 478 bp long region. Since no individual belonged to H63/H65/H66/H67/H79 haplotypes, these sequences were removed from the database to limit the risk of collapsing different haplotypes and thus reducing the informativity. The definitive COI haplotype inference was redone on a 592 bp long region. A ΔybgF reference database for haplotype inference was created with the sequences published by Otero‐Bravo & Sabree (2018) and Martinez‐Sañudo *et al.* (2020) (Table S4).

For all newly identified *H. halys* markers, haplotype name “_h1” was given to the corresponding sequence deposited in the reference genome (GCF_000696795). For all newly identified *P. carbekii* markers haplotype name “_h1” was given to the corresponding sequence deposited in the genome NZ_CP010907, whilst “_h2” to the corresponding sequence deposited in the genome AP012554.

Number of haplotypes (H), haplotype diversity (h) and nucleotide diversity (π) were calculated with R package pegas version 1.3 (Paradis, 2010). Haplotype networks were generated with TCS version 1.23 (Clement *et al.*, 2000) and graphically displayed with tcsBU (Múrias dos Santos *et al.*, 2016). The association between host and symbiont molecular markers was assessed using the specialization index H2’ (Blüthgen *et al.*, 2006) and visualized as bipartite network using R package bipartite (Dormann *et al.*, 2008) as described by Martinez‐Sañudo *et al.* (2020).

## Results

### Halyomorpha halys mitochondrial markers

Amplicons were obtained for all 72 individuals for COI and for 71 individuals for COII. A total of 11 different COI haplotypes (Figs. S1A and S2–S4) were detected by analysing sequences from 71 individuals, with three of them never recorded before (individuals from China). The novel haplotypes were named H168, H169 and H170 (Fig. S1A) (Acc. Nos.: PQ643351–643353). Except for populations from Reggio Emilia (IT) and Samsun (TR), all the other populations showed at least two different COI haplotypes. The most abundant haplotype was H1 (40 sequences), followed by H3 and H43 (6 sequences). Haplotypes H169 and H170 were present in a single individual (Table S5). The π values varied from a minimum of 0 to a maximum of 0.00409, while h values from a minimum of 0 to a maximum of 0.844 ± 0.068. In both cases, the lowest values belonged to the invasive populations of Reggio Emilia (IT) and Samsun (TR), while the highest values to the native population of Henan (CN) (Table 2). By grouping the populations for their Country, the greater number of different haplotypes found was displayed by Chinese samples (H = 8) (Table 2).

**Table 2:** Summary statistics for each population under analysis using markers available in literature

	COI				COII				ΔybgF			
Province‐country	*n*	H	π	h ± SD	*n*	H	π	h ± SD	*n*	H	π	h ± SD
Cuneo‐IT	9	5	0.00347	0.806 ± 0.113	10	2	0.00042	0.200 ± 0.157	7	2	0.00139	0.286 ± 0.200
Reggio Emilia‐IT	11	1	0	0 ± 0	8	1	0	0 ± 0	4	1	0	0 ± 0
Cremona‐IT	10	5	0.00379	0.867 ± 0.058	8	2	0.00091	0.429 ± 0.166	8	2	0.00208	0.428 ± 0.166
Samsun‐TR	8	1	0	0 ± 0	8	1	0	0 ± 0	7	1	0	0 ± 0
Ordu‐TR	13	2	0.00048	0.282 ± 0.143	13	2	0.00060	0.282 ± 0.143	11	2	0.00088	0.182 ± 0.146
Beijing‐CN	10	4	0.00154	0.644 ± 0.150	10	4	0.00183	0.711 ± 0.111	9	2	0.00270	0.556 ± 0.070
Henan‐CN	10	5	0.00409	0.844 ± 0.068	10	3	0.00141	0.600 ± 0.125	10	3	0.00194	0.378 ± 0.184
**Italy**	30	5	0.00388	0.729 ± 0.062	26	2	0.00107	0.508 ± 0.036	19	2	0.00238	0.491 ± 0.064
**Türkiye**	21	2	0.00031	0.181 ± 0.105	21	2	0.00038	0.181 ± 0.105	18	2	0.00054	0.111 ± 0.098
**China**	20	8	0.00336	0.847 ± 0.057	20	5	0.00157	0.632 ± 0.086	19	3	0.00250	0.485 ± 0.103

*Note*: COI and COII: *H. halys* mitochondrial markers; ΔybgF: *P. carbekii* marker. Results are given separately for the provinces where samples were collected (plain font) and for the countries under investigation (bold font): Italy comprehends samples from Cuneo, Reggio Emilia, and Cremona; Türkiye samples are from Samsun and Ordu; China samples from Beijing and Henan. *n*: number of sequences; H: number of haplotypes; π: nucleotide diversity; h: haplotype diversity.

Five different haplotypes were identified for COII (473 bp) (Figs. S1B and S2–S4) out from 67 high quality sequences obtained, with a newly described one named h22 from Henan (CN) (one sequence; Table S5) (Acc. No.: PQ658198). Similarly to COI, no haplotype diversity was found in Reggio Emilia (IT) and Samsun (TR) populations. In general, the most abundant haplotype was h1 (41 sequences), followed by h3 (23 sequences). All other haplotypes (h5, n15, h22) were found in single individuals. Similar to COI, the least variable populations were the invasive ones of Reggio Emilia (IT) and Samsun (TR) (π = 0; h = 0), while the Chinese population of Beijing showed the greatest π (0.00183) and h (0.711 ± 0.111) values (Table 2).

### Halyomorpha halys nuclear markers

**Fig. 1:**
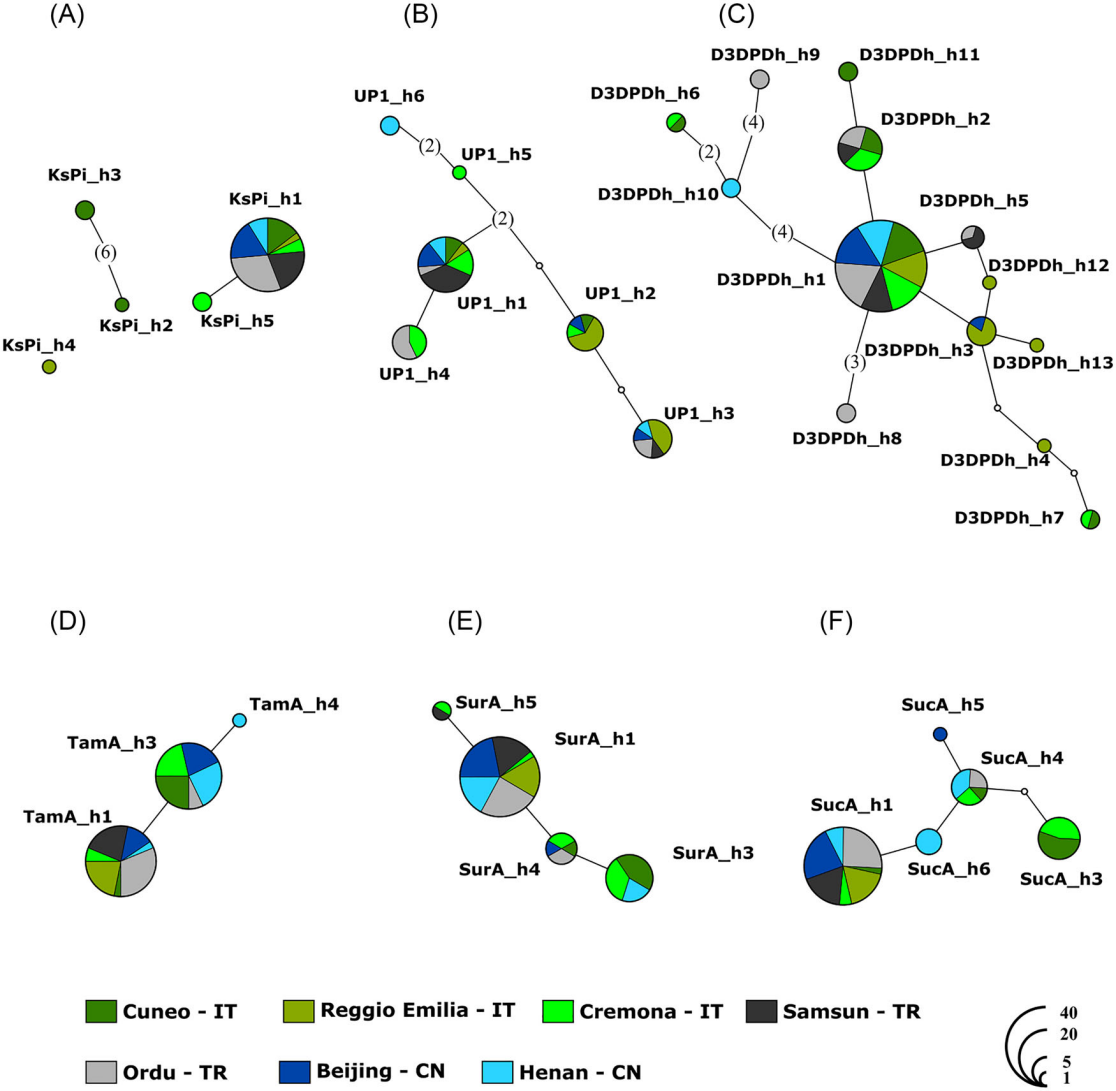
Haplotype distribution according to newly identified *H. halys* nuclear markers and “*Candidatus* Pantoea carbekii” markers along the sampled populations. Circle size increases with the number of samples belonging to each haplotype. Small white circles represent putative or missing haplotypes forecasted by the analysis. Numbers in round brackets represent the number of consecutive putative/missing haplotypes present in the graph. (A) Hh_KsPi haplotypes, (B) Hh_UP1 haplotypes, (C) Hh_D3PDh haplotypes, (D) Pc_TamA haplotypes, (E) Pc_SurA haplotypes, (F) Pc_SucA haplotypes.

Haplotype inference for Hh_KsPi was performed on a 950 bp region, and amplicons were obtained for all individuals. A total of 40 sequences were obtained out of 72 individuals analyzed. From the remaining 32 individuals, amplicons were obtained but the resulting sequences showed overlapping peaks after a few high‐quality bases, indicating heterozygosity. In most cases, a difference in the electrophoretic mobility was clear, with not perfectly overlapping double peaks. When allele inference was performed on remaining electropherograms, many indels and SNPs resulted between generated and reference sequences, thus indicating a high risk of artifact generation. For these reasons, sequences generated from heterozygous individuals were discarded from the analysis. A total of five distinct haplotypes were identified (Fig. 1A) (Hh_KsPi_h1–Hh_KsPi_h5 Acc. Nos.: PQ658212–PQ658216). Polymorphism with reference genome sequence ranged from 1 SNP of KsPi_h5 to 36 SNPs and six indels of KsPi_h4. Details on number and type of polymorphism detected among new haplotypes and reference genome can be found in Table S6. All haplotypes different from Hh_KsPi_h1 (which was found in all tested populations) were detected from Italian samples and all Italian populations exhibited at least two different haplotypes (Table 3; Figs. 1A and S2–S4). Chinese and Turkish populations only showed KsPi_h1 haplotype, thus resulting in π and h values equal to 0 (Table 3; Figs. 1A and S2–S4).

**Table 3:** Summary statistics for each population under analysis according to the newly identified markers

Province‐country	*n*	H	π	h ± SD	*n*	H	π	h ± SD	*n*	H	π	h ± SD
	Hh_KsPi				Hh_UP1				Hh_D3PDh			
Cuneo‐IT	8	3	0.01045	0.607 ± 0.160	3	2	0.00343	0.667 ± 0.272	15	5	0.00555	0.695 ± 0.108
Reggio Emilia‐IT	2	2	0.04255	1	10	3	0.00377	0.644 ± 0.090	14	5	0.00236	0.703 ± 0.098
Cremona‐IT	4	2	0.00075	0.667 ± 0.144	8	4	0.00376	0.786 ± 0.098	13	4	0.00550	0.654 ± 0.102
Samsun‐TR	7	1	0	0 ± 0	8	2	0.00257	0.250 ± 0.184	10	3	0.00173	0.622 ± 0.135
Ordu‐TR	10	1	0	0 ± 0	7	3	0.00637	0.667 ± 0.150	18	5	0.00864	0.673 ± 0.105
Beijing‐CN	6	1	0	0 ± 0	5	3	0.00514	0.700 ± 0.208	9	2	0.00054	0.222 ± 0.170
Henan‐CN	3	1	0	0 ± 0	5	3	0.01080	0.800 ± 0.129	9	2	0.00473	0.389 ± 0.164
**Italy**	14	5	0.01269	0.670 ± 0.126	21	5	0.00524	0.786 ± 0.042	42	9	0.00469	0.697 ± 0.068
**Türkiye**	17	1	0	0 ± 0	15	3	0.00460	0.648 ± 0.084	28	5	0.00626	0.643 ± 0.086
**China**	9	1	0	0 ± 0	10	4	0.00811	0.733 ± 0.114	18	3	0.00281	0.307 ± 0.133
	Pc_TamA				Pc_SurA				Pc_SucA			
Cuneo‐IT	8	2	0.00037594	0.250 ± 0.184	7	2	0.000541126	0.286 ± 0.200	8	3	0.00223951	0.464 ± 0.202
Reggio Emilia‐IT	7	1	0	0 ± 0	7	1	0	0 ± 0	7	1	0	0 ± 0
Cremona‐IT	8	2	0.000644468	0.429 ± 0.167	9	4	0.002209596	0.694 ± 0.143	9	3	0.003116978	0.667 ± 0.125
Samsun‐TR	7	1	0	0 ± 0	8	2	0.000473485	0.250 ± 0.184	7	1	0	0 ± 0
Ordu‐TR	12	2	0.000455685	0.303 ± 0.149	12	2	0.000573921	0.303 ± 0.149	12	2	0.0010001	0.303 ± 0.149
Beijing‐CN	10	2	0.000802005	0.533 ± 0.081	10	2	0.000378788	0.200 ± 0.157	10	2	0.000990099	0.200 ± 0.157
Henan‐CN	9	3	0.000668338	0.417 ± 0.193	10	2	0.001767677	0.467 ± 0.127	10	3	0.001540154	0.733 ± 0.059
**Italy**	23	2	0.000772683	0.514 ± 0.035	23	4	0.002096059	0.660 ± 0.060	24	3	0.003384034	0.627 ± 0.048
**Türkiye**	19	2	0.000298993	0.199 ± 0.113	20	3	0.000548246	0.279 ± 0.125	19	2	0.000656206	0.199 ± 0.113
**China**	19	3	0.000773864	0.485 ± 0.103	20	3	0.001146332	0.353 ± 0.124	20	4	0.001554629	0.605 ± 0.100

*Note*: Hh_KsPi, Hh_UP1, Hh_D3PDh: *H. halys* nuclear markers; Pc_TamA, Pc_SurA, Pc_SucA: *P. carbekii* markers. Results are given separately for the provinces where samples were collected (plain font) and for the countries under investigation (bold font): Italy comprehends samples from Cuneo, Reggio Emilia, and Cremona; Türkiye samples are from Samsun and Ordu; China samples from Beijing and Henan. *n*: number of sequences; H: number of haplotypes; π: nucleotide diversity; h: haplotype diversity.

Haplotype inference for Hh_UP1 was performed on a 392 bp region, resulting in 41 sequences from 72 individuals analyzed. Nine of them were obtained as one allele inferred with Indigo. Given the presence of both SNPs and indels in this marker (Table S6), other inferred alleles that resulted as singleton were discarded from the analysis to minimize the risk of generating artifactual sequences. All the individuals showed proper amplification and multiple haplotypes were identified in each population analyzed, for a total of six different haplotypes (Figs. 1B and S2–S4). The most prevalent haplotype was UP1_h1 (19 sequences) (Acc. No.: PQ658217) followed by UP1_h3 (9 sequences) (Acc. No.: PQ658218) and UP1_h2 (8 sequences) (Acc. No.: PQ658219) (Figs. 1B and S2–S4). Only UP1_h5 (Acc. No.: PQ658221) was present as a single copy in a homozygous individual (Fig. 1B). Polymorphisms with reference genome sequence ranged from one SNP of UP1_h4 (Acc. No.: PQ658220) to five SNPs and one base deletion of UP1_h6 (Acc. No.: PQ658222) (Table S6). Both π and h were higher in populations from invaded areas compared to what described by mitochondrial markers (Table 3).

Also for Hh_D3PDh amplicons were obtained for all 72 *H. halys* individuals. Haplotype inference was performed on a 411 bp region, yielding 88 sequences, 48 of which were inferred. Since most of the polymorphisms between the alleles were SNPs, and only a one bp indel was present, allele inference was more successful for this marker. Details on number and type of polymorphism can be found in Table S6. A total of 13 haplotypes were identified, with multiple haplotypes detected in each analyzed population (Figs. 1C and S2–S4). Populations of invaded areas showed overall higher haplotype diversity compared to native ones (Table 3). The dominant haplotype was D3PDh_h1 haplotype (53 sequences) (Acc. No.: PQ658199), followed by D3PDh_h2 (12 sequences) (Acc. No.: PQ658200), and three haplotypes were identified as a single copy (D3PDh_h4, D3PDh_h12 and D3PDh_h13) (Acc. Nos.: PQ658202, PQ658210, PQ658211). Indel polymorphisms involved a single base deletion in haplotypes D3PDh_h4, D3PDh_h7 (Acc. No.: PQ658205) and D3PDh_h8 (Acc. No.: PQ658206) compared to reference genome. SNP polymorphisms ranged from one SNP (haplotypes D3PDh_h2, D3PDh_h3 and D3PDh_h5) to a maximum of 12 SNPs (haplotype D3PDh_h9) (Hh_D3PDh_h3/5/9 Acc. Nos.: PQ658201, PQ658203, PQ658207). Sequences corresponding to haplotypes Hh_D3PDh_h6/10/11 can be retrieved with Acc. Nos.: PQ658204, PQ658208, PQ658209. Italian populations generally showed higher π values than when using mitochondrial markers. In Turkish populations, both π and h were generally higher compared to mitochondrial markers. Chinese populations, instead, showed low π and h values (Table 3).

### “Candidatus Pantoea carbekii” markers

Amplicons of ΔybgF were obtained for 64 out of 66 individuals. Three different haplotypes were identified by analyzing 56 ΔybgF sequences (Figs. S1C and S2–S4; Table S5). Two of them had been previously described, namely P1 and P2; these haplotypes were nearly equally distributed, with 29 and 26 sequences, respectively. The third one was a novel haplotype, named P8 (Acc. No.: PQ666878), found in a single individual from the Henan population (CN) (Fig. S4). The highest haplotype diversity (three different haplotypes) was identified in the Henan population, while only one haplotype was found in the invasive populations of Reggio Emilia (IT) and Samsun (TR). In the other populations, both P1 and P2 were present.

Among the newly developed markers, amplicons were obtained for 64 out of 66 individuals for Pc_TamA marker. Haplotype inference was performed on a 665 bp region, revealing three different haplotypes (Figs. 1D and S2–S4). Information obtained by this marker was redundant with ΔybgF in invasive populations (IT–TR), with all the individuals having P1 haplotype displaying TamA_h1 (Acc. No: PQ659259) and individuals having P2 haplotype displaying TamA_h3 (Acc. No: PQ659260). This was not true for native individuals (CN), where one individual having P8 haplotype displayed TamA_h3 and one individual having P2 haplotype displayed TamA_h4 (Acc. No.: PQ659261) (Table S5). No indel with reference genome corresponding sequence was found in this or in any other newly identified markers. The most common haplotype was TamA_h1 (32 sequences), followed by TamA_h3 (28 sequences) (Fig. 1D). Haplotype TamA_h3 differed by one SNP from the corresponding sequence in reference genome, while TamA_h4 by two SNPs (Table S6). DNA mutations resulted in amino acids missense with TamA_h1 haplotype (Table S7). For both TamA_h3 and TamA_h4 the first non‐synonymous mutation led to the substitution of a Lysine with an Asparagine, while the second TamA_h4 to the substitution of a Methionine with an Isoleucine. The h values were similar to the ones for ΔybgF, whilst π values were generally lower due to the greater length of TamA marker compared to ΔybgF (Table 3).

Four different Pc_SurA haplotypes were identified in a 528 bp region (Figs. 1E and S2–S4). Proper amplification was obtained for all individuals. All four haplotypes were identified in Italian individuals, while three haplotypes were found in Chinese and Turkish individuals (Figs. 1E and S2–S4). SurA_h3 (Acc. No.: PQ659256) haplotype showed two SNPs with reference genome sequence (Acc. No.: PQ659255), while SurA_h4 and SurA_h5 (Acc. Nos.: PQ659257, PQ659258) had a single SNP (Table S6), with all these SNPs resulting in amino acids substitutions (Table S7). The first SurA_h3 SNP was shared with SurA_h4 haplotype, resulting in the substitution of a Leucine with a Proline, while the second SurA_h3 SNP resulted in the substitution of an Asparagine with an Aspartic Acid. The single SNP of SurA_h5 haplotype was not shared with any other haplotype and resulted in the substitution of a Valine with a Glycine (Table S7). Unlike for the other *P. carbekii* markers, two haplotypes of Pc_SurA were identified in Samsun (TR) population. Higher h values were described for this marker in invaded areas compared to ΔybgF (Table 3).

A total of 64 amplicons were obtained for Pc_SucA. This marker was the most variable marker of *P. carbekii*, with five different haplotypes identified in a 606 bp region (Figs. 1F and S2–S4). Interestingly, only three of them namely SucA_h1, SucA_h3 and SucA_h4 (Acc. Nos: PQ659250, PQ659251, PQ659252) were found in invasive populations (IT and TR), making this marker the most informative only for the native populations (Henan‐CN and Beijing‐CN) (Fig. S4). The most abundant haplotype was SucA_h1, present in 39 individuals along all the populations under analysis. SucA_h3 and SucA_h4 were almost equally represented, with respectively eleven and eight individuals, but SucA_h3 was only present in Cuneo (IT) and Cremona (IT) populations, whilst SucA_h4 was found also in Ordu (TR) and Henan (CN) populations. SucA_h6 was found only in four samples of the Henan (CN) population, while SucA_h5 (Acc. No.: PQ659253) in a single individual from Beijing (CN) population. SucA_h6 (Acc. No.: PQ659254), SucA_h4/h5/h3 showed 1, 2, 3 and 4 SNPs, respectively, compared to the reference genome sequence (Table S6). Not all SNPs led to missense amino acids, with just two missense changes in total (Table S7). The only SNP of SucA_h6 led to the substitution of an Isoleucine with a Valine, shared with all the other haplotypes. SucA_h3, SucA_h4 and SucA_h5 SNPs resulted in the substitution of Glutamic acid with a Lysine. Also for this marker, higher h values were observed in invaded areas compared to ΔybgF (Table 3).

By comparing putative protein structures, even in the cases where mutations led to amino acids with different properties (Table S7), no structural damage was showed by inferring protein 3D structure for any of the markers studied.

### MLST analysis

**Fig. 2:**
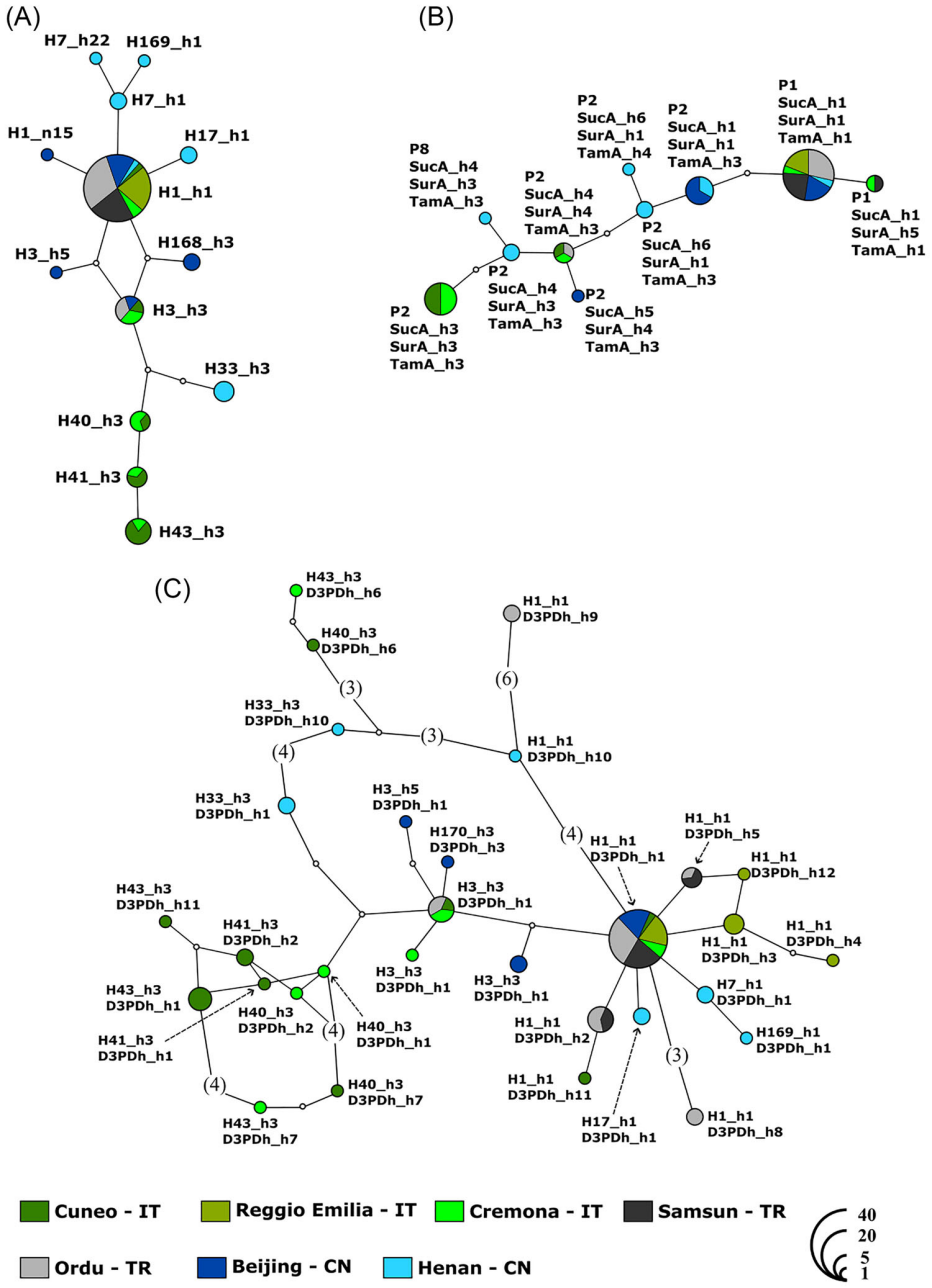
Haplotype distribution according to MLST analyses. Circle size increases with the number of samples belonging to each haplotype. Small white circles represent putative or missing haplotypes forecasted by the analysis. Numbers in round brackets represent the number of consecutive putative/missing haplotypes present in the graph. (A) COI+COII markers, (B) ΔybgF + Pc_SucA + Pc_SurA + Pc_TamA markers, (C) COI + COII + Hh_D3PDh markers.

The combination of COI and COII sequences led to the identification of 13 different haplotypes from 66 individuals (Fig. 2A). Compared with COI, only in the native populations of Beijing (CN) and Henan (CN) the combination with COII increased the number of described haplotypes. This is confirmed by h value, which increased only for Chinese populations (Table 4). In Reggio Emilia (IT) and Samsun (TR) populations, mitochondrial genes identified only a single haplotype (Fig. 2A; Table 4).

**Table 4:** Summary statistics for each population under analysis using different MLST approaches

Province‐Country	*n*	H	π	h ± SD	*n*	H	π	h ± SD
			COI + COII				*P. carbekii*	
Cuneo‐IT	9	5	0.00214	0.806 ± 0.113	5	2	0.00060	0.400 ± 0.239
Reggio Emilia‐IT	8	1	0	0	4	1	0	0
Cremona‐IT	8	5	0.00252	0.893 ± 0.067	7	4	0.00219	0.714 ± 0.177
Samsun‐TR	8	1	0	0	6	2	0.00017	0.333 ± 0.219
Ordu‐TR	13	2	0.00053	0.282 ± 0.143	8	2	0.00062	0.250 ± 0.184
Beijing‐CN	10	5	0.00148	0.756 ± 0.126	9	3	0.00010	0.667 ± 0.091
Henan‐CN	10	6	0.00290	0.889 ± 0.065	9	6	0.00144	0.917 ± 0.058
**Italy**	25	5	0.00267	0.753 ± 0.062	16	4	0.00211	0.675 ± 0.082
**Türkiye**	21	2	0.00034	0.181 ± 0.105	14	3	0.00043	0.275 ± 0.151
**China**	20	10	0.00247	0.890 ± 0.048	18	7	0.00135	0.824 ± 0.058
		COI + COII + D3PDh				COI + COII + D3PDh + *P. carbekii*		
Cuneo‐IT	13	9	0.00332	0.910 ± 0.064	6	3	0.00086	0.600 ± 0.213
Reggio Emilia‐IT	10	4	0.00065	0.711 ± 0.111	4	3	0.00034	0.833 ± 0.188
Cremona‐IT	9	7	0.00384	0.944 ± 0.057	8	8	0.00297	1
Samsun‐TR	10	3	0.00048	0.622 ± 0.135	8	4	0.00032	0.821 ± 0.084
Ordu‐TR	18	6	0.00269	0.778 ± 0.080	12	6	0.00119	0.758 ± 0.120
Beijing‐CN	9	4	0.00117	0.694 ± 0.143	8	5	0.00116	0.857 ± 0.096
Henan‐CN	9	6	0.00343	0.917 ± 0.058	8	8	0.00219	1
**Italy**	32	17	0.00342	0.917 ± 0.032	18	12	0.00260	0.935 ± 0.037
**Türkiye**	28	6	0.00193	0.717 ± 0.075	20	7	0.00084	0.763 ± 0.078
**China**	18	10	0.00254	0.909 ± 0.046	16	13	0.00190	0.967 ± 0.031

*Note*: Results are given separately for the provinces where samples were collected (plain font) and for the countries under investigation (bold font): Italy comprehends samples from Cuneo, Reggio Emilia, and Cremona; Türkiye samples are from Samsun and Ordu; China samples from Beijing and Henan. *P. carbekii* = ΔybgF + Pc_TamA + Pc_SucA + Pc_SurA markers. D3PDh = Hh_D3PDh marker. *n*: number of sequences; H: number of haplotypes; π: nucleotide diversity; h: haplotype diversity.

Using all four *P. carbekii* markers, the total number of symbiont haplotypes identified out of 48 samples raised from three (obtained using only ΔybgF) to 10 (Fig. 2B). In line with the host MLST, all populations exhibited at least two different haplotypes, except for that from Reggio Emilia (IT). The higher variability was described for Henan (CN) and Cremona (IT) populations, with six and four haplotypes, respectively. For each population analyzed, h values increased compared to those obtained with ΔybgF alone (Table 4).

An MLST approach incorporating both mitochondrial and the best‐performing nuclear marker (i.e., Hh_D3PDh) was also tested, yielding a total of 78 sequences (Fig. 2C). Compared to the use of COI + COII, the number of described *H. halys* haplotypes increased from 13 to 30 (Fig. 2A, C). Not only native populations, but also invasive populations displayed a higher number of haplotypes. Italian and Turkish populations increased from five and two COI + COII haplotypes to 17 and 6, respectively. For all populations analyzed, the h value sensibly increased (Table 4).

A holobiont approach, combining the best performing *H. halys* makers with those from *P. carbekii*, was also tested. By using 52 sequences coming from COI + COII + Hh_D3PDh + the four *P. carbekii* markers, 28 joint *H. halys* + *P. carbekii* haplotypes were identified (Fig. S5). For Henan (CN) and Cremona (IT) populations, h value reached 1 (Table 4).

Finally, the use of the second‐best performing *H. halys* nuclear marker (i.e., Hh_UP1) along with mitochondrial and with both mitochondrial and symbiont sequences was assessed (Fig. S6, Table S8). For the combination of mitochondrial markers and Hh_UP1, 41 sequences were used. Compared to the use of COI + COII the number of identified haplotypes increased from 13 to 17 (Fig. S6). Despite a general increase in the number of scored haplotypes, in Cuneo (IT) population the number of haplotypes decreased from five to three (Table S8). This reduction resulted from the lack of UP1 sequences for seven individuals of Cuneo (IT) population (Table S5). By combining COI + COII + Hh_UP1 + the four *P. carbekii* markers only 25 sequences were available, describing 17 different haplotypes (Fig. S6, Table S8). Except for Cuneo (IT) and the two Turkish populations of Samsun and Ordu, h value reached 1 (Table S8).

### Host and symbiont haplotypes associations

**Fig. 3:**
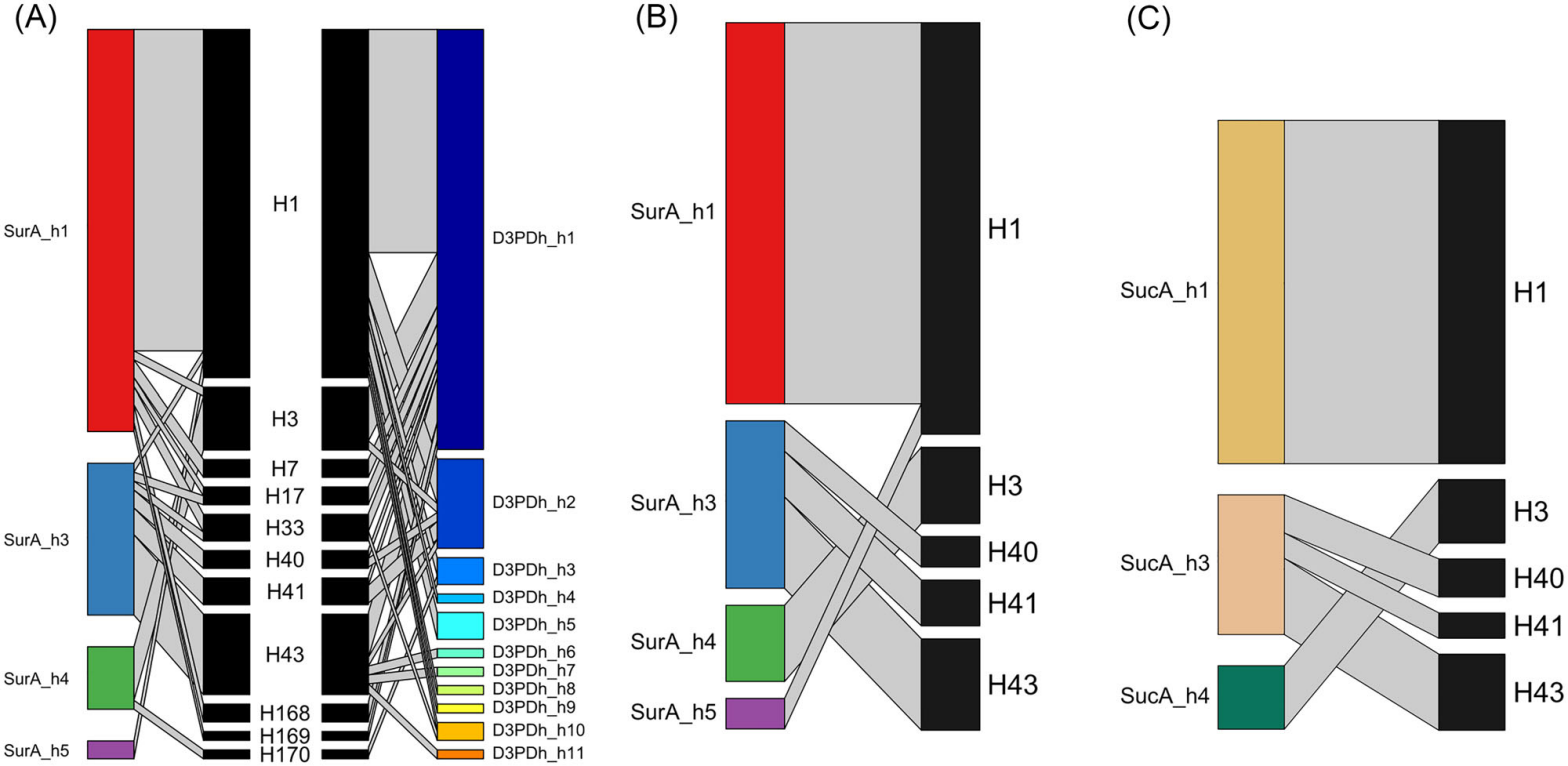
Bipartite networks of interaction between *H. halys* COI haplotypes and novel nuclear and “*Candidatus* Pantoea carbekii” markers haplotypes. The width of the conjunctions represents the frequency with which two haplotypes were found in a same individual. Different colours of the rectangles represent different haplotypes of the used marker. (A) Comparison between Pc_SurA–COI and Hh_D3PDh–COI bipartite interactions, (B) bipartite network between Pc_SurA and COI in invasive populations, (C) bipartite network between Pc_SucA and COI in invasive populations.

The specialization index H2’ was calculated for the association between COI and COII, COI and the two most informative *P. carbekii* markers (Pc_SurA and Pc_SucA), COI and the two most informative nuclear markers (Hh_D3PDh and Hh_UP1), and between the most informative nuclear and *P. carbekii* markers. The highest network specializations were shown between the two mitochondrial markers (COI–COII H2’ = 0.850) and between COI and the *P. carbekii* markers (COI–Pc_SucA H2’ = 0.799; COI–Pc_SurA H2’ = 0.662). In contrast, Hh_D3PDh showed the lowest H2’ values, both in relation to COI (COI–Hh_D3PDh H2’ = 0.047) and *P. cabekii* markers (Hh_D3PDh–Pc_SucA H2’ = 0.163; Hh_D3PDh–Pc_SurA H2’ = 0.081). Moreover, Hh_UP1 demonstrated low network specialization, both with COI (COI–Hh_UP1 H2’ = 0.275) and with the *P. cabekii* markers (Hh_UP1–Pc_SucA H2’ = 0.210; Hh_UP1–Pc_SurA H2’ = 0.097). Differences in network specialization between COI with Hh_D3PDh and COI with Pc_SurA are shown in Fig. 3A. Bipartite interaction matrices for other combinations of markers described in this paragraph are shown in Supplementary material (Figs. S7 and S8). Interestingly, by considering only the invasive populations (Cuneo‐IT, Reggio Emilia‐IT, Cremona‐IT, Samsun‐TR and Ordu‐TR), a perfect network specialization was found between COI and the most informative *P. carbekii* markers (COI–Pc_SucA H2’ = 1; COI–Pc_SurA H2’ = 1) (Fig. 3B, C).

## Discussion

### Mitochondrial markers

The use of molecular markers to reconstruct *H. halys* invasion patterns has been widely used over the last decade (Xu *et al.*, 2014; Cesari *et al.*, 2015; Zhu *et al.*, 2016; Valentin *et al.*, 2017; Cesari *et al.*, 2018; Kapantaidaki *et al.*, 2019; Gariepy *et al.*, 2021; Laterza *et al.*, 2023; Berteloot *et al.*, 2024). However, defining invasion routes by using one or a few genetic markers has become progressively more challenging due to the occurrence of both primary and secondary invasions, as well as the growing number of different haplotypes in invaded areas over time (Valentin *et al.*, 2017; Cesari *et al.*, 2018; Schuler *et al.*, 2021; Berteloot *et al.*, 2024).

In this study, by sequencing a 592 bp region of the COI gene of 71 individuals, 11 different haplotypes were identified. From Beijing (CN) population, two novel haplotypes, H168 and H170, were discovered. Haplotype network analysis revealed that H168 is a variant of the widespread H1 haplotype, while H170 is a variant of H3, each differing from their known counterparts by a single SNP. Both H1 and H3 are well documented and abundant in Chinese populations (Gariepy *et al.*, 2015; Valentin *et al.*, 2017; Lee *et al.*, 2018; Martinez‐Sañudo *et al.*, 2020), and many haplotypes differing by one single SNP from H1 (e.g., H12, H15, H18, H20) or from H3 (e.g., H9, H11, H21, H22) have been described before (Gariepy *et al.*, 2014; Cesari *et al.*, 2015; Valentin *et al.*, 2017). By increasing the number of analyzed samples in native areas it is thus expected to identify more variants of the most abundantly represented haplotypes. A new haplotype, H169, was also identified in an individual from Henan (CN) population, differing for one SNP from the less common H7 haplotype, previously detected in both China and California (Valentin *et al.*, 2017; Lee *et al.*, 2018). Interestingly, two individuals of the H3 haplotype were detected in Ordu (TR) population, making the first instance of a haplotype other than H1 being recorded in Türkiye. This result suggests that either two separate invasion events occurred in the country, or that this haplotype was not previously detected due to its lower abundance (Yan *et al.*, 2021a). The origin of Turkish populations suggested by COI could be either primary (i.e. from China) or secondary, with Italy being a likely source. Additionally, other haplotypes were identified for the first time in a certain invaded region. For instance, haplotype H40 was newly detected in Cremona (IT) although it had already been present in Cuneo province (IT), suggesting a secondary invasion event (Cesari *et al.*, 2018). These novel haplotype detections may result from the rarity of these haplotypes, which previous studies may have missed due to limited sample size, or they may indicate actual novel invasions. Five haplotypes were identified from COII sequences, including the newly described h22 haplotype, which differs from the common h1 for one SNP, found in a single individual from Henan (CN) population. The presence in Ordu (TR) population of h1 and h3 haplotypes found in both Italian and Chinese populations suggests these two areas as putative sources. Interestingly, the combined use of COI with the less variable COII, commonly employed to increase haplotype resolution (Cesari *et al.*, 2018; Yan *et al.*, 2021a; Berteloot *et al.*, 2024), was more informative than COI alone for the native populations only (Fig. 2A). This finding aligns with a previous study demonstrating that the combined use of these two markers did not outperform COI alone in tracking the origin of *H. halys* populations invasive in Belgium (Berteloot *et al.*, 2024). Moreover, the high network specialization between these two markers (H2’ = 0.850) confirms that COII and COI haplotypes tend to be associated. These results suggest that markers other than COII might be more effective to further explore *H. halys* genetic diversity, also according to the occurrence of other, more variable, mitochondrial regions, reported by Gogniashvili *et al.* (2021).

The reduced number of individuals retained for this combined analysis (66 out of 72) resulted from the fact that not for every stink bug high quality sequences were obtained for both markers. (Table S5). As the number of sequenced loci increases, the risk of not obtaining high‐quality sequences for each marker in every individual also increases. Since even single miscalled SNPs can result in different haplotype attributions, unclear sequences were discarded, leading to a reduced number of individuals retained for MLST approaches in general. Increasing informativeness by adding molecular markers comes with a higher risk of reducing the number of samples retained for the analysis (Cesari *et al.*, 2018; Berteloot *et al.*, 2024).

### Symbiont markers

Since vertically transmitted endosymbionts genomes tend to coevolve with the host (Funk *et al.*, 2000), symbiont markers have been proposed to reconstruct the movement of invasive insect species (El Sheikha & Menozzi, 2019). However, the *P. carbekii* ΔybgF marker, so far known in seven haplotypes, has shown to be not variable enough to be used for properly reconstructing the phylogeography of the host (Otero‐Bravo & Sabree, 2018; Martinez‐Sañudo *et al.*, 2020). In this study, the novel P8 haplotype was identified in a single individual from Henan population, but the overall low variability of the marker was reaffirmed. To address this insufficiency, three novel markers were developed in this study by exploiting *P. carbekii* annotated genome. Except for Pc_TamA, these markers exhibited greater variability than ΔybgF. Interestingly, even though five different haplotypes were identified for Pc_SucA, only three were found in invaded areas (SucA_h1/h3/h4). On the other hand, Pc_SurA revealed four haplotypes, all of which were present in invasive populations (Fig. 1F). Notably, in the case of Samsun (TR) population, Pc_SurA was more informative than COI, identifying two haplotypes instead of only one. The presence in this population of a higher genetic variability than the one described by COI only is confirmed by analyzing *H. halys* nuclear markers Hh_UP1 and Hh_D3PDh (Fig. 1B, C). Only with all four symbiont markers an optimal characterization of the symbiont is achieved since each one explains part of the genetic variability unrevealed by the others. This approach led to the identification of 10 *P. carbekii* haplotypes in 48 individuals (Table S5), two more than those previously identified using ΔybgF alone (Fig. 2B). The presence of two shared haplotypes between Italian and Turkish populations (P2 – SucA_h4 – SurA_h4 – TamA_h3; P1 – SucA_h1 – SurA_h5 – TamA_h1) and only one common to all countries (P1 – SucA_h1 – SurA_h1 – TamA_h1) (Fig. 2B) suggests that a secondary invasion event, from Italy to Türkiye, could be the source of Turkish populations. To confirm this hypothesis, however, detailed studies with larger sampling size and using these newly described markers are needed. No indels were observed in the haplotypes of the newly identified *P. carbekii* markers compared to the reference genome, which is expected given that all these markers were derived from coding regions. Interestingly, in several cases, SNPs were related to missense mutation, even with the presence of amino acids characterized by different properties (Table S7). However, protein structure predictions showed no structural damage, suggesting that these coding gene variations are not detrimental to *P. carbekii*; to confirm this, the full sequence of the gene is required. These findings suggest that *P. carbekii* exhibits greater genetic variability than previously assumed, which may enhance its potential as a tool for tracing the origin of *H. halys* invasive populations. The functional implication of the recorded variability is still to be determined, but we cannot exclude that symbiont variants are involved in different insect performances, with additional consequences on invasive capability.

### MLST: host mitochondrial and symbiont markers

In a previous study, seven *P. carbekii* haplotypes associated with 25 COI *H. halys* haplotypes were identified (Martinez‐Sañudo *et al.*, 2020). In the current study, by combining the four *P. carbekii* markers mentioned above, 10 bacterial haplotypes were found to be associated with 11 *H. halys* COI haplotypes. In accordance with the findings of Martinez‐Sañudo *et al.* (2020), the H2’ value highlights a strong network specialization between *H. halys* COI haplotypes and those of the symbiont, even when considering more informative markers such as Pc_SucA and Pc_SurA. As expected for two separate genomes, the specialization between *H. halys* COI and *P. carbekii* markers is lower than the one between COI and COII. This suggests that using COI in combination with *P. carbekii* markers may yield more informative results than using COI and COII. Interestingly, the result changes drastically when only invasive populations are considered. In these populations, the perfect network specialization between COI and the symbiont markers strengthens the hypothesis that certain host–symbiont variants may possess enhanced invasive capabilities (Martinez‐Sañudo *et al.*, 2020) (Fig. 3B, C). However, further studies are required to understand if this perfect specialization is just the result of the genetic bottleneck that invasive populations normally undergo (Tsutsui *et al.*, 2000; Puillandre *et al.*, 2008; Croft *et al.*, 2024), or if these host–symbiont variants have an advantage in invading new areas compared to other haplotype combinations. Furthermore, it would be valuable to investigate whether specific functional variants in the genome of *P. carbekii* can contribute to increased host adaptability.

### Nuclear markers

The variability of one or few mitochondrial markers can be insufficient to clearly define the invasion routes. Alternative strategies such as the use of the whole mitochondrial sequence (Morón‐López *et al.*, 2022) or the joint use of mitochondrial and nuclear markers to overcome the limit of only matrilineal inheritance (Li *et al.*, 2015) proved to be more effective in population genetics studies and invasion events reconstruction. Thus, several authors suggested to expand the use of *H. halys* nuclear markers to overcome the lack of clear genetic discontinuity between areas identified by mitochondrial markers (Valentin *et al.*, 2017; Cesari *et al.*, 2018). For this purpose, in this study, three novel nuclear markers have been identified. The best performing nuclear marker was Hh_D3PDh. Since only one indel of one nucleotide was present between the haplotypes, this marker suits well for allele inference process with bioinformatic tools such as Tracy (Rausch *et al.*, 2020). The lower π and h values observed in native populations compared to invasive ones could result from continuous inbreeding occurring in the laboratory‐reared colony (Beijing–CN) (Table 3). Another reason could be related to the ongoing primary and secondary invasion events occurring in invaded areas, as described in Italy (Cesari *et al.*, 2018; Schuler *et al.*, 2021). The concomitant presence of haplotypes normally separated in native areas can lead to genetic admixture (Parvizi *et al.*, 2023).

Interestingly, the low H2’ value obtained for both Hh_D3PDh and Hh_UP1 indicates very low network specialization between nuclear markers and both mitochondrial and symbiont markers. This result confirms the great potential of nuclear markers in adding informativity to the analyses. As an example, with only mitochondrial markers all the Turkish individuals shared haplotypes with both Italian and Chinese populations (Fig. S1A, B). By using Hh_D3PDh and Hh_UP1, the haplotypes D3PDh_h2 and UP1_h4 were retrieved only from Italian and Turkish individuals (Fig. 1B, C). This result could suggest a secondary invasion event as the source of Turkish populations. Given the limited number of samples, further studies focusing on *H. halys* movements and invasion pathway reconstruction are needed to confirm this hypothesis.

However, for almost half of the individuals tested (32 for Hh_KsPi; 32 for Hh_D3PDh; 38 for Hh_UP1), heterozygosity was observed in these markers’ gene regions and for Hh_KsPi the discrimination between alleles was unreliable due to the presence of multiple SNPs and indels. An NGS‐based approach applied to the proposed nuclear targets could allow surpassing heterozygosity issues. The relatively short regions corresponding to both Hh_D3PDh and Hh_UP1 markers make them suitable for high‐throughput short‐read sequencing, thus eliminating the need for allele inference, while Hh_KsPi marker, corresponding to a longer gene region, could potentially better describe individual variability due to the concurrent presence of multiple indels and SNPs.

In this study, novel *H. halys* nuclear markers and *P. carbekii* markers have been identified. These markers are expected to be valuable tools for population genetics studies and shed light on the invasion sources of *H. halys* populations, helping in preventing or limiting outbreaks. Since the use of multiple markers tends to increase the risk of losing some individuals from the analysis (Cesari *et al.*, 2018; Yan *et al.*, 2021a; Berteloot *et al.*, 2024), the selection of a limited number of reliable and variable markers is pivotal to increase the informativity of the analysis, keeping it cost‐effective and not time consuming. For these reasons, a sort of “top‐down MLST” approach could be preferable. A first screening with the widely used COI would help in defining putative source regions. A further discrimination with *P. carbekii* markers such as Pc_SurA or Pc_SucA could help narrowing the range of possible sources. The addition of nuclear markers could eventually help in further limiting the possible spectrum when previous markers don't give an unambiguous answer. Hh_D3PDh is preferable when Sanger sequencing methods are used, while Hh_UP1 and Hh_KsPi suit better high throughput sequencing. Future efforts to strengthen this approach should be done to create larger databases with sequences for the here proposed markers from both native and invaded areas.

## Disclosure

The authors have declared that no competing interests exist.

## Supporting information




**Table S1** Details of target sequences, primer used for amplification, and annealing temperature of markers used for *Halyomorpha halys* and “*Candidatus* Pantoea carbekii” haplotyping.

**Table S2** List of accession numbers of *Halyomorpha halys* COI haplotypes used for haplotype inference.

**Table S3** List of accession numbers of *Halyomorpha halys* COII haplotypes used for haplotype inference.

**Table S4** List of accession numbers of “*Candidatus* Pantoea carbekii” ΔYbgF haplotypes used for haplotype inference.

**Table S5** List of haplotypes identified for each marker under analysis and for each sample.

**Table S6** Summary of polymorphisms among different haplotypes and reference genomes for the newly identified molecular markers.

**Table S7** Amino acid missenses resulting from the comparison between newly identified “*Candidatus* Pantoea carbekii” haplotypes and h1, corresponding to reference genome (Acc. Num.: NZ_CP010907.1).

**Table S8** Summary statistics for each population under analysis with different MLST approaches using Hh_UP1 as nuclear marker.

**Fig. S1** Haplotype distribution according to *H. halys* mitochondrial markers and “*Ca*. Pantoea carbekii” ΔybgF along the sampled populations.

**Fig. S2** Haplotypes found for each marker under analysis in Italian populations.

**Fig. S3** Haplotypes found for each marker under analysis in Turkish populations.

**Fig. S4** Haplotypes found for each marker under analysis in Chinese populations.

**Fig. S5** Haplotype distribution obtained combining the most informative markers from *Halyomorpha halys* and “*Candidatus* Pantoea carbekii” in the populations under analysis.

**Fig. S6** Haplotype distribution obtained using different MLST approaches.

**Fig. S7** Bipartite interaction matrices between *Halyomorpha halys* and “*Candidatus* Pantoea carbekii” haplotypes for all the populations under analysis.

**Fig. S8** Bipartite networks of interaction between haplotypes of the “*Candidatus* Pantoea carbekii” Pc_SurA marker and *Halyomorpha halys* COI marker (left side), and between *H. halys* COI and Hh_UP1 markers (right side).

## References

[ins70034-bib-0001] Abram, P.K. , Hueppelsheuser, T. , Acheampong, S. , Clarke, P. , Douglas, H. and Gariepy, T.D. (2017) Evidence of established brown marmorated stink bug populations in British Columbia, Canada. Journal of the Entomological Society of British Columbia, 114, 83–86.

[ins70034-bib-0002] Arnold, K. (2009) *Halyomorpha halys* (Stål, 1855), eine für die europäische Fauna neu nachgewiesene Wanzenart (Insecta: Heteroptera: Pentatomidae: Cappaeini). Mitteilungen Des Thüringer Entomologenverbandes, 16, e19.

[ins70034-bib-0003] Artimo, P. , Jonnalagedda, M. , Arnold, K. , Baratin, D. , Csardi, G. , De Castro, E. *et al.* (2012) ExPASy: SIB bioinformatics resource portal. Nucleic Acids Research, 40, W597–W603. 22661580 10.1093/nar/gks400 PMC3394269

[ins70034-bib-0004] Bansal, R. , Michel, A.P. and Sabree, Z.L. (2014) The crypt‐dwelling primary bacterial symbiont of the polyphagous pentatomid pest *Halyomorpha halys* (Hemiptera: Pentatomidae). Environmental Entomology, 43, 617–625. 24874153 10.1603/EN13341

[ins70034-bib-0005] Bariselli, M. , Bugiani, R. and Maistrello, L. (2016) Distribution and damage caused by *Halyomorpha halys* in Italy. EPPO Bulletin, 46, 332–334.

[ins70034-bib-0006] Berteloot, O.H. , Kuhn, A. , Peusens, G. , Beliën, T. , Hautier, L. , Van Leeuwen, T. *et al.* (2024) Distribution and genetic diversity of the invasive pest *Halyomorpha halys* (Hemiptera, Pentatomidae) in Belgium. NeoBiota, 90, 123–138.

[ins70034-bib-0007] Blüthgen, N. , Menzel, F. and Blüthgen, N. (2006) Measuring specialization in species interaction networks. BMC Ecology, 6, 9. 16907983 10.1186/1472-6785-6-9 PMC1570337

[ins70034-bib-0008] Bosco, L. , Moraglio, S.T. and Tavella, L. (2018) *Halyomorpha halys*, a serious threat for hazelnut in newly invaded areas. Journal of Pest Science, 91, 661–670.

[ins70034-bib-0009] Callot, H. and Brua, C. (2013) *Halyomorpha halys* (Stål, 1855), la Punaise diabolique, nouvelle espèce pour la faune de France (Heteroptera Pentatomidae). L'Entomologiste, 69, 69–71.

[ins70034-bib-0010] Cesari, M. , Maistrello, L. , Ganzerli, F. , Dioli, P. , Rebecchi, L. and Guidetti, R. (2015) A pest alien invasion in progress: potential pathways of origin of the brown marmorated stink bug *Halyomorpha halys* populations in Italy. Journal of Pest Science, 88, 1–7.

[ins70034-bib-0011] Cesari, M. , Maistrello, L. , Piemontese, L. , Bonini, R. , Dioli, P. , Lee, W. *et al.* (2018) Genetic diversity of the brown marmorated stink bug *Halyomorpha halys* in the invaded territories of Europe and its patterns of diffusion in Italy. Biological Invasions, 20, 1073–1092.

[ins70034-bib-0012] Clement, M. , Posada, D. and Crandall, K. (2000) TCS: a computer program to estimate gene genealogies. Molecular Ecology, 9, 1657–1659. 11050560 10.1046/j.1365-294x.2000.01020.x

[ins70034-bib-0013] Croft, L. , Matheson, P. , Butterworth, N.J. and McGaughran, A. (2024) Fitness consequences of population bottlenecks in an invasive blowfly. Molecular Ecology, 33, e17492. 39136044 10.1111/mec.17492

[ins70034-bib-0014] Damos, P. , Soulopoulou, P. and Thomidis, T. (2019) Establishment and current status of *Halyomorpha halys* damaging peaches and olives in the prefecture of Imathia in Northern Greece. International Organization for Biological and Integrated Control, IOBC, West Palearctic Regional Section (WPRS) Bulletin, 146, 111–113.

[ins70034-bib-0015] Dho, M. , Gonella, E. and Alma, A. (2025) Field evaluation of symbiont‐targeted control of *Halyomorpha halys* in hazelnut crop. Crop Protection, 187, 106952. 10.3390/insects16070688 PMC12294827 40725318

[ins70034-bib-0016] Dormann, C.F. , Gruber, B. and Fründ, J. (2008) Introducing the bipartite package: Analysing ecological networks. Interaction, 8, 8–11.

[ins70034-bib-0017] El Sheikha, A.F. and Menozzi, P. (2019) Potential geo‐tracing tool for migrant insects by using 16S rDNA fingerprinting of bacterial communities by PCR‐DGGE. International Journal of Tropical Insect Science, 39, 9–16.

[ins70034-bib-0018] Emms, D.M. and Kelly, S. (2019) OrthoFinder: Phylogenetic orthology inference for comparative genomics. Genome Biology, 20, 238. 31727128 10.1186/s13059-019-1832-y PMC6857279

[ins70034-bib-0019] Faúndez, E.I. and Rider, D.A. (2017) The brown marmorated stink bug *Halyomorpha halys* (Stål, 1855) (Heteroptera: Pentatomidae) in Chile. Arquivos Entomolóxicos, 17, 305–307.

[ins70034-bib-0020] Ficetola, G.F. , Bonin, A. and Miaud, C. (2008) Population genetics reveals origin and number of founders in a biological invasion. Molecular Ecology, 17, 773–782. 18194168 10.1111/j.1365-294X.2007.03622.x

[ins70034-bib-0021] Funk, D.J. , Helbling, L. , Wernegreen, J.J. and Moran, N.A. (2000) Intraspecific phylogenetic congruence among multiple symbiont genomes. Proceedings of the Royal Society of London. Series B: Biological Sciences, 267, 2517–2521. 10.1098/rspb.2000.1314 PMC1690841 11197128

[ins70034-bib-0022] Gandjaeva, L.A. , Hudayberdieva, M.O. , Abdullaev, I.I. , Mirzayeva, G.S. and Yusupboev, E.K. (2022) First record of *Halyomorpha halys* (Heteroptera: Pentatomidae) from Uzbekistan. Zoosystematica Rossica, 31, 329–331.

[ins70034-bib-0023] Gariepy, T.D. , Bruin, A. , Haye, T. , Milonas, P. and Vétek, G. (2015) Occurrence and genetic diversity of new populations of *Halyomorpha halys* in Europe. Journal of Pest Science, 88, 451–460.

[ins70034-bib-0024] Gariepy, T.D. , Haye, T. , Fraser, H. and Zhang, J. (2014) Occurrence, genetic diversity, and potential pathways of entry of *Halyomorpha halys* in newly invaded areas of Canada and Switzerland. Journal of Pest Science, 87, 17–28.

[ins70034-bib-0025] Gariepy, T.D. , Musolin, D.L. , Konjević, A. , Karpun, N.N. , Zakharchenko, V.Y. , Zhuravleva, E.N. *et al.* (2021) Diversity and distribution of cytochrome oxidase I (COI) haplotypes of the brown marmorated stink bug, *Halyomorpha halys* Stål (Hemiptera, Pentatomidae), along the eastern front of its invasive range in Eurasia. NeoBiota, 68, 53–77.

[ins70034-bib-0026] Gaspar, H. , Castro, S. , Grosso‐Silva, J. , van der Heyden, T. and Loureiro, J. (2023) Exponential outspread of *Halyomorpha halys* (Stål, 1855) (Hemiptera: Pentatomidae) in Portugal. Arquivos Entomolóxicos, 22, 373–376.

[ins70034-bib-0027] Gogniashvili, M. , Kunelauri, N. , Shanava, T. , Tephnadze, N. and Beridze, T. (2021) Complete nucleotide sequence of the mitochondrial DNA of *Halyomorpha halys* (Stål) (Hemiptera: Pentatomidae) specimens collected across Georgia. Advances in Entomology, 9, 113–121.

[ins70034-bib-0028] Gonella, E. and Alma, A. (2023) The role of symbiont‐targeted strategies in the management of Pentatomidae and Tephritidae pests under an integrated vision. Agronomy, 13, 868.

[ins70034-bib-0029] Hancock, T.J. , Lee, D. , Bergh, J.C. , Morrison, W.R. and Leskey, T.C. (2019) Presence of the invasive brown marmorated stink bug *Halyomorpha halys* (Stål) (Hemiptera: Pentatomidae) on home exteriors during the autumn dispersal period: results generated by citizen scientists. Agricultural and Forest Entomology, 21, 99–108.

[ins70034-bib-0030] Heckmann, R. (2012) Erster nachweis von *Halyomorpha halys* (Stål, 1855) (Heteroptera: Pentatomidae) für Deutschland. Heteropteron, 36, 17–18.

[ins70034-bib-0031] Hoebeke, E.R. and Carter, M.E. (2003) *Halyomorpha halys* (Stål) (Heteroptera: Pentatomidae): a polyphagous plant pest from Asia newly detected in North America. Proceedings of the Entomological Society of Washington, 105, 225–237.

[ins70034-bib-0032] Horwood, M. , Milnes, J.M. and Cooper, W.R. (2019) Brown marmorated stink bug, *Halyomorpha halys* (Hemiptera: Pentatomidae), detections in Western Sydney, New South Wales, Australia. Austral Entomology, 58, 857–865.

[ins70034-bib-0033] Ittisoponpisan, S. , Islam, S.A. , Khanna, T. , Alhuzimi, E. , David, A. and Sternberg, M.J. (2019) Can predicted protein 3D structures provide reliable insights into whether missense variants are disease associated? Journal of Molecular Biology, 431, 2197–2212. 30995449 10.1016/j.jmb.2019.04.009 PMC6544567

[ins70034-bib-0034] Kalashian, M.Y. , Ghrejyan, T. and Karagyan, G. (2022) Brown marmorated stink bug *Halyomorpha halys* (Stål, 1855) (Heteroptera: Pentatomidae) penetrated into Armenia. Russian Journal of Biological Invasions, 13, 305–308.

[ins70034-bib-0035] Kapantaidaki, D.E. , Evangelou, V.I. , Morrison, W.R. , Leskey, T.C. , Brodeur, J. and Milonas, P. (2019) *Halyomorpha halys* (Hemiptera: Pentatomidae) genetic diversity in North America and Europe. Insects, 10, 174. 31212913 10.3390/insects10060174 PMC6628459

[ins70034-bib-0036] Kenyon, L.J. , Meulia, T. and Sabree, Z.L. (2015) Habitat visualization and genomic analysis of “*Candidatus* Pantoea carbekii,” the primary symbiont of the brown marmorated stink bug. Genome Biology and Evolution, 7, 620–635. 25587021 10.1093/gbe/evv006 PMC4350177

[ins70034-bib-0037] Laterza, I. , Sabree, Z.L. , Martinez‐Sañudo, I. , Scaccini, D. , Pozzebon, A. , Cornara, D. *et al* (2023) *Halyomorpha halys* in Mediterranean areas: Local and landscape predictors, genetic diversity, and potential biological control. Entomologia Generalis, 43, 981–990.

[ins70034-bib-0038] Lee, D.‐H. , Short, B.D. , Joseph, S.V. , Bergh, J.C. and Leskey, T.C. (2013) Review of the biology, ecology, and management of *Halyomorpha halys* (Hemiptera: Pentatomidae) in China, Japan, and the Republic of Korea. Environmental Entomology, 42, 627–641. 23905725 10.1603/EN13006

[ins70034-bib-0039] Lee, W. , Guidetti, R. , Cesari, M. , Gariepy, T. , Park, Y.L. and Park, C.G. (2018) Genetic diversity of *Halyomorpha halys* (Hemiptera, Pentatomidae) in Korea and comparison with COI sequence datasets from East Asia, Europe, and North America. Florida Entomologist, 101, 49–54.

[ins70034-bib-0040] Leskey, T.C. and Nielsen, A.L. (2018) Impact of the invasive brown marmorated stink bug in North America and Europe: history, biology, ecology, and management. Annual Review of Entomology, 63, 599–618. 10.1146/annurev-ento-020117-043226 29068708

[ins70034-bib-0041] Leskey, T.C. , Short, B.D. , Butler, B.R. and Wright, S.E. (2012) Impact of the invasive brown marmorated stink bug, *Halyomorpha halys* (Stål), in mid‐Atlantic tree fruit orchards in the United States: case studies of commercial management. Psyche: A Journal of Entomology, 2012, e535062.

[ins70034-bib-0042] Li, Y. , Guo, X. , Chen, L. , Bai, X. , Wei, X. , Zhou, X. *et al.* (2015) Inferring invasion history of Red Swamp Crayfish (*Procambarus clarkii*) in China from mitochondrial control region and nuclear intron sequences. International Journal of Molecular Sciences, 16, 14623–14639. 26132567 10.3390/ijms160714623 PMC4519862

[ins70034-bib-0043] Maistrello, L. , Dioli, P. , Vaccari, G. , Nannini, R. , Bortolotti, P. , Caruso, S. *et al*. (2014) Primi rinvenimenti in Italia della cimice esotica Halyomorpha halys, una nuova minaccia per la frutticoltura. ATTI Giornate Fitopatologiche, 1, 283–288.

[ins70034-bib-0044] Martinez‐Sañudo, I. , Perotti, M.A. , Scaccini, D. , Pozzebon, A. , Marri, L. and Mazzon, L. (2020) Co‐haplotyping symbiont and host to unravel invasion pathways of the exotic pest *Halyomorpha halys* in Italy. Scientific Reports, 10, 18441. 33116256 10.1038/s41598-020-75519-2 PMC7595193

[ins70034-bib-0045] Medina, R.F. , Barbosa, P. , Christman, M. and Battisti, A. (2006) Number of individuals and molecular markers to use in genetic differentiation studies. Molecular Ecology Notes, 6, 1010–1013.

[ins70034-bib-0046] Milonas, P.G. and Partsinevelos, G.K. (2014) First report of brown marmorated stink bug *Halyomorpha halys* Stål (Hemiptera: Pentatomidae) in Greece. EPPO Bulletin, 44, 183–186.

[ins70034-bib-0047] Morey, A.C. , Kerzicnik, L.M. , Etzler, F.E. , Mendrey, K. , Morey, B.D. and Miller, Z. (2022) First report of *Halyomorpha halys* (Hemiptera: Pentatomidae) in Montana, USA. Journal of Integrated Pest Management, 13, 27.

[ins70034-bib-0048] Morón‐López, J. , Vergara, K. , Sato, M. , Gajardo, G. and Ueki, S. (2022) Intraspecies variation of the mitochondrial genome: An evaluation for phylogenetic approaches based on the conventional choices of genes and segments on mitogenome. PLoS ONE, 17, e0273330. 35980990 10.1371/journal.pone.0273330 PMC9387813

[ins70034-bib-0049] Múrias dos Santos, A. , Cabezas, M.P. , Tavares, A.I. , Xavier, R. and Branco, M. (2016) tcsBU: a tool to extend TCS network layout and visualization. Bioinformatics, 32, 627–628. 26515821 10.1093/bioinformatics/btv636

[ins70034-bib-0050] Nouere, S. , Amiri, S. and Lahlali, R. (2020) Situation des problèmes phytosanitaires du myrtillier (*Vaccinium corymbosum*) au Maroc. Revue Marocaine Des Sciences Agronomiques Et Vétérinaires, 8, 321–330.

[ins70034-bib-0051] Otero‐Bravo, A. and Sabree, Z.L. (2018) Comparing the utility of host and primary endosymbiont loci for predicting global invasive insect genetic structuring and migration patterns. Biological Control, 116, 10–16.

[ins70034-bib-0052] Paradis, E. (2010) Pegas: an R package for population genetics with an integrated–modular approach. Bioinformatics, 26, 419–420. 20080509 10.1093/bioinformatics/btp696

[ins70034-bib-0053] Parvizi, E. , Dhami, M.K. , Yan, J. and McGaughran, A. (2023) Population genomic insights into invasion success in a polyphagous agricultural pest, *Halyomorpha halys* . Molecular Ecology, 32, 138–151. 36261398 10.1111/mec.16740 PMC10099481

[ins70034-bib-0054] Petrovan, S. , Aldridge, D. , Smith, R. , White, T. and Sutherland, W. (2022) *Halyomorpha halys* invasion front jumps 1500 kilometres to reach the Canary Islands; a framework for rapid response, identification of urgent questions, and assessment of potential impacts. ARPHA Preprints, 3, e84924.

[ins70034-bib-0055] Pistone, D. , Mugu, S. and Jordal, B.H. (2016) Genomic mining of phylogenetically informative nuclear markers in Bark and Ambrosia Beetles. PLoS ONE, 11, e0163529. 27668729 10.1371/journal.pone.0163529 PMC5036811

[ins70034-bib-0056] Puillandre, N. , Dupas, S. , Dangles, O. , Zeddam, J.‐L. , Capdevielle‐Dulac, C. , Barbin, K. *et al.* (2008) Genetic bottleneck in invasive species: the potato tuber moth adds to the list. Biological Invasions, 10, 319–333.

[ins70034-bib-0057] Rausch, T. , Fritz, M.H.Y. , Untergasser, A. and Benes, V. (2020) Tracy: basecalling, alignment, assembly and deconvolution of sanger chromatogram trace files. BMC Genomics, 21, 230. 32171249 10.1186/s12864-020-6635-8 PMC7071639

[ins70034-bib-0058] Rice, K.B. , Bergh, C.J. , Bergmann, E.J. , Biddinger, D.J. , Dieckhoff, C. , Dively, G. *et al.* (2014) Biology, ecology, and management of brown marmorated stink bug (Hemiptera: Pentatomidae). Journal of Integrated Pest Management, 5, A1–A13.

[ins70034-bib-0059] Rius, M. , Bourne, S. , Hornsby, H.G. and Chapman, M.A. (2015) Applications of next‐generation sequencing to the study of biological invasions. Current Zoology, 61, 488–504.

[ins70034-bib-0060] Rozas, J. , Ferrer‐Mata, A. , Sánchez‐DelBarrio, J.C. , Guirao‐Rico, S. , Librado, P. , Ramos‐Onsins, S.E. *et al*. (2017) DnaSP 6: DNA sequence polymorphism analysis of large data sets. Molecular Biology and Evolution, 34, 3299–3302. 29029172 10.1093/molbev/msx248

[ins70034-bib-0061] Schuler, H. , Elsler, D. and Fischnaller, S. (2021) Population genetics of the brown marmorated stink bug *Halyomorpha halys* in the early phase of invasion in South Tyrol (Northern Italy). Bulletin of Entomological Research, 111, 394–401. 33106194 10.1017/S0007485320000553

[ins70034-bib-0062] Suyama, M. , Torrents, D. and Bork, P. (2006) PAL2NAL: Robust conversion of protein sequence alignments into the corresponding codon alignments. Nucleic Acids Research, 34, W609–W612. 16845082 10.1093/nar/gkl315 PMC1538804

[ins70034-bib-0063] Tamura, K. , Stecher, G. and Kumar, S. (2021) MEGA11: molecular evolutionary genetics analysis version 11. Molecular Biology and Evolution, 38, 3022–3027. 33892491 10.1093/molbev/msab120 PMC8233496

[ins70034-bib-0064] Temreshev, I.I. , Esenbekova, P.A. and Uspanov, A.M. (2018) New Records of a dangerous invasive pests−brown marmorated stink bug *Halyomorpha halys* Stål, 1855 (Heteroptera, Pentatomidae) in Kazakhstan. Acta Biologica Sibirica, 4, 94.

[ins70034-bib-0065] Tsutsui, N.D. , Suarez, A.V. , Holway, D.A. and Case, T.J. (2000) Reduced genetic variation and the success of an invasive species. Proceedings of the National Academy of Sciences USA, 97, 5948–5953. 10.1073/pnas.100110397 PMC18539 10811892

[ins70034-bib-0066] Valentin, R.E. , Nielsen, A.L. , Wiman, N.G. , Lee, D.H. and Fonseca, D.M. (2017) Global invasion network of the brown marmorated stink bug. Halyomorpha Halys. Scientific Reports, 7, 9866. 28852110 10.1038/s41598-017-10315-z PMC5575200

[ins70034-bib-0067] van der Heyden, T. , Saci, A. and Dioli, P. (2021) First record of the brown marmorated stink bug *Halyomorpha halys* (Stål, 1855) in Algeria and its presence in North Africa (Heteroptera: Pentatomidae). Revista Gaditana De Entomología, 12, 147–154.

[ins70034-bib-0068] Wermelinger, B. , Wyniger, D. and Forster, B. (2007) First records of an invasive bug in Europe: *Halyomorpha halys* Stål (Heteroptera: Pentatomidae), a new pest on woody ornamentals and fruit trees? Mitteilungen Der Schweizerischen Entomologischen Gesellschaft, 81, 1–8.

[ins70034-bib-0069] Xu, J. , Fonseca, D.M. , Hamilton, G.C. , Hoelmer, K.A. and Nielsen, A.L. (2014) Tracing the origin of US brown marmorated stink bugs, *Halyomorpha halys* . Biological Invasions, 16, 153–166.

[ins70034-bib-0070] Yan, J. , Pal, C. , Anderson, D. , Vétek, G. , Farkas, P. , Burne, A. *et al.* (2021a) Genetic diversity analysis of brown marmorated stink bug, *Halyomorpha halys* based on mitochondrial COI and COII haplotypes. BMC Genomic Data, 22, 7. 33588747 10.1186/s12863-021-00961-8 PMC7885415

[ins70034-bib-0071] Yan, J. , Vétek, G. , Pal, C. , Zhang, J. , Gmati, R. , Fan, Q.H. *et al.* (2021b) ddRAD sequencing: An emerging technology added to the biosecurity toolbox for tracing the origin of brown marmorated stink bug, *Halyomorpha halys* (Hemiptera: Pentatomidae). BMC Genomics, 22, 355. 34000993 10.1186/s12864-021-07678-z PMC8130256

[ins70034-bib-0072] Zhu, G.P. , Ye, Z. , Du, J. , Zhang, D.L. , Zhen, Y. , Zheng, C. *et al.* (2016) Range wide molecular data and niche modeling revealed the Pleistocene history of a global invader (*Halyomorpha halys*). Scientific Reports, 6, 23192. 26996353 10.1038/srep23192 PMC4800403

