# Supplementary material for: Multilocus sequence typing of the invasive pest Halyomorpha halys (Hemiptera: Pentatomidae) and associated endosymbiont reveals unexplored diversity
Source: Insect Sci. 2025 Apr 9;33(3):1187–205. doi: 10.1111/1744-7917.70034 (PMC13252635; doi:10.1111/1744-7917.70034)
Supplement: Supplementary file 1 — Table S1 Details of target sequences, primer used for amplification, and annealing temperature of markers used for Halyomorpha halys and “Candidatus Pantoea carbekii” haplotyping. [file INS-33-1187-s008.docx]

| **Organism** | **Target** | **Primer name** | **Primer sequence (5’-3’)** | **Annealing Temp.** | **Amplicon position (bp)** | **Reference. seq.** | **Corresponding gene** |
| --- | --- | --- | --- | --- | --- | --- | --- |
| *Haltomorpha halys* (mitochondrion) | COI | Hh_Alt-COI-F | CTAAAAATTTCAGCCACTTTA | 52.0°C | 1366-2159 | LC579925.1 | Cytochrome c oxidase subunit I |
|  |  | Hh_Alt-COI-R | TATGGGAAATTAAGCCAAATC |  |  |  |  |
|  | COII | HhalysCO2altF | TTTTAACCCAAGATGCAAATTC | 48.0°C | * | * | Cytochrome c oxidase subunit II |
|  |  | HhalysCO2altR | TTGTTCTTTCAATTACGATTGG |  |  |  |  |
| *Halyomorpha halys* (nucleus) | D3PDh | Hh_D3PDh-F | TAGGGCAGTCAACATTCGGC | 59.5 °C | 36173-36666 | NW_020111215.1 | D-3-phosphoglycerate dehydrogenase |
|  |  | Hh_D3PDh-R | AAAACGTCCGTTTGTGCTGG |  |  |  |  |
|  | UP1 | Hh_UP1-F | CGCCATGTAGGAAGGTCCTC | 60.0°C | 307699-308198 | NW_020110311.1 | Uncharacterized prot LOC106692253 |
|  |  | Hh_UP1-R | TCTTTCGTGGGTGACTCTGC |  |  |  |  |
|  | KsPi | KsPi_intron_2-F | GGCAACGCTTCCACAATCTG | 58.5°C | 336427-337443 | NW_020110557.1 | Kazal-type serine protease inhibitor domain-containing |
|  |  | KsPi_intron_2-R | TAGACTGCGAAGCCAAAGGG |  |  |  |  |
| *‘Candidatus* Pantoea carbekii*’* | ΔYbgF | ΔYbg-F | TAGTGCAAGCAGTCGT | 50.0°C | ** | ** | Pseudogene |
|  |  | ΔYbg-R | GAGTATCTTGCTTATGTTGC |  |  |  |  |
|  | SucA | Pc_SucA-F | AAGCTAGGTTTTCTGCGCCT | 57.2°C | 543436-544123 | NZ_CP010907.1 | 2-oxoglutarate dehydrogenase  E1 component |
|  |  | Pc_SucA-R | ATGTCACAAGCTCGCGGTTA |  |  |  |  |
|  | TamA | Pc_TamA-F | CTCTTGTTACAAAGGGCTCAACA | 58.6°C | 399277-400025 | NZ_022547.1 | Autotransporter assembly  complex prot TamA |
|  |  | Pc_TamA-R | GACGCGGTGATACAAAAGCA |  |  |  |  |
|  | SurA | Pc_SurA-F | GAAGCATTACAAGATGGCAGCA | 58.6°C | 666772-667372 | NC_022547.1 | Peptidylprolyl isomerase SurA |
|  |  | Pc_SurA-R | TCACATAAGCCATGCCTCGTT |  |  |  |  |

Multi locus sequence typing of the invasive pest *Halyomorpha halys* and associated endosymbiont reveals unexplored diversity

Matteo Dho^1^, Matteo Montagna^2^, Chenxi Liu^3^, Alberto Alma^1^, Elena Gonella^1*^

**Supplementary materials**

**Table S1.** Details of target sequences, primer used for amplification, and annealing temperature of markers used for *Halyomorpha halys* and ‘*Candidatus* Pantoea

carbekii*’* haplotyping. Asterisks indicate markers found in literature, specifically * = Cesari et al., 2018. **= Otero-Bravo and Sabree, 2018.

**Table S7.** Amino acid missenses resulting from the comparison between newly identified *‘Candidatus* Pantoea carbekii*’* haplotypes and h1, corresponding to reference genome (Acc. Num.: NZ_CP010907.1). To avoid frameshift, the flanking 5’ and 3’ regions were substituted with the sequence present in the reference genome before translation.

| **Marker** | **Haplotype** | **Amino acid change** | **Position in primary structure** | **Amino acid property** |
| --- | --- | --- | --- | --- |
| Pc_TamA | TamA_h3/h4 | Lys 🡪 Asn | 85 | + charged 🡪 polar aa |
|  | TamA_h4 | Met 🡪 Ile | 131 | Both non-polar |
| Pc_SurA | SurA_h5 | Val 🡪 Gly | 282 | Both non-polar |
|  | SurA_h3/h4 | Leu 🡪 Pro | 293 | Both non-polar |
|  | SurA_h3 | Asn 🡪 Asp | 384 | Polar 🡪 - charged aa |
| Pc_SucA | SucA_h3/h4/h5/h6 | Ile 🡪 Val | 436 | Both non-polar |
|  | SucA_h3/h4/h5 | Glu 🡪 Lys | 520 | - charged 🡪 + charged aa |

**Table S8.** Summary statistics for each population under analysis with different MLST approaches using Hh_UP1 as nuclear marker. Results are given separately for the regions where samples were collected and for the countries under investigation: Italy comprehends samples from Cuneo, Reggio Emilia and Cremona; Turkey samples are from Samsun and Ordu; China samples from Beijing and Henan. *P. carbekii* = ΔybgF + Pc_TamA + Pc_SucA + Pc_SurA markers. n: number of sequences; H: number of haplotypes; π: nucleotide diversity; h: haplotype diversity.

| **Province - Country** | **n** | **H** | **π** | **h ± SD** | **n** | **H** | **π** | **h ± SD** |
| --- | --- | --- | --- | --- | --- | --- | --- | --- |
|  | **COI + COII + UP1** | | | | **COI + COII + UP1+ *P. cabekii*** | | | |
| Cuneo – IT | 3 | 3 | 0.00321 | 1 | 0 | 0 | 0 | 0 |
| Reggio Emilia – IT | 7 | 2 | 0.00066 | 0.476±0.165 | 2 | 2 | 0.00058 | 1 |
| Cremona – IT | 6 | 6 | 0.00275 | 1 | 5 | 5 | 0.00237 | 1 |
| Samsun – TR | 8 | 2 | 0.00069 | 0.250±0.184 | 6 | 3 | 0.00048 | 0.600±0.213 |
| Ordu – TR | 7 | 3 | 0.00210 | 0.667±0.150 | 4 | 2 | 0.00072 | 0.500±0.258 |
| Beijing – CN | 5 | 5 | 0.00248 | 1 | 4 | 4 | 0.00174 | 1 |
| Henan – CN | 5 | 5 | 0.00523 | 1 | 4 | 4 | 0.00313 | 1 |
| **Italy** | 16 | 9 | 0.00285 | 0.858±0.075 | 7 | 7 | 0.00278 | 1 |
| **Turkey** | 15 | 4 | 0.00142 | 0.714±0.076 | 10 | 4 | 0.00060 | 0.778±0.080 |
| **China** | 10 | 9 | 0.00404 | 0.978±0.043 | 8 | 8 | 0.00267 | 1 |


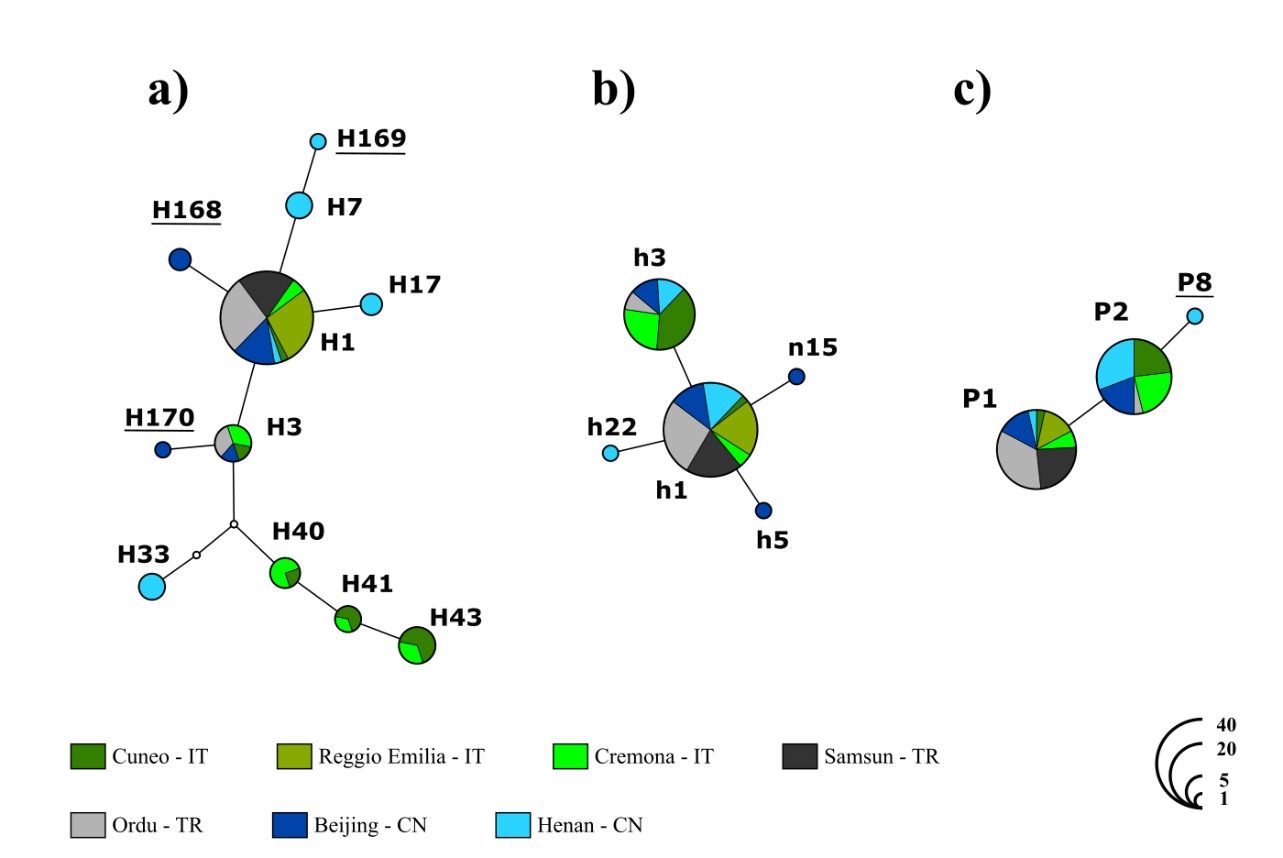


**Figure S1.** Haplotype distribution according to *H. halys* mitochondrial markers and *‘Ca.* Pantoea carbekii’ ΔybgF along the sampled populations. Circle size increases with the number of samples belonging to each haplotype. Small white circles represent putative or missing haplotypes forecasted by the analysis. Underlined haplotypes are described for the first time. **A** COI haplotypes **B** COII haplotypes **C** ΔybgF haplotypes.


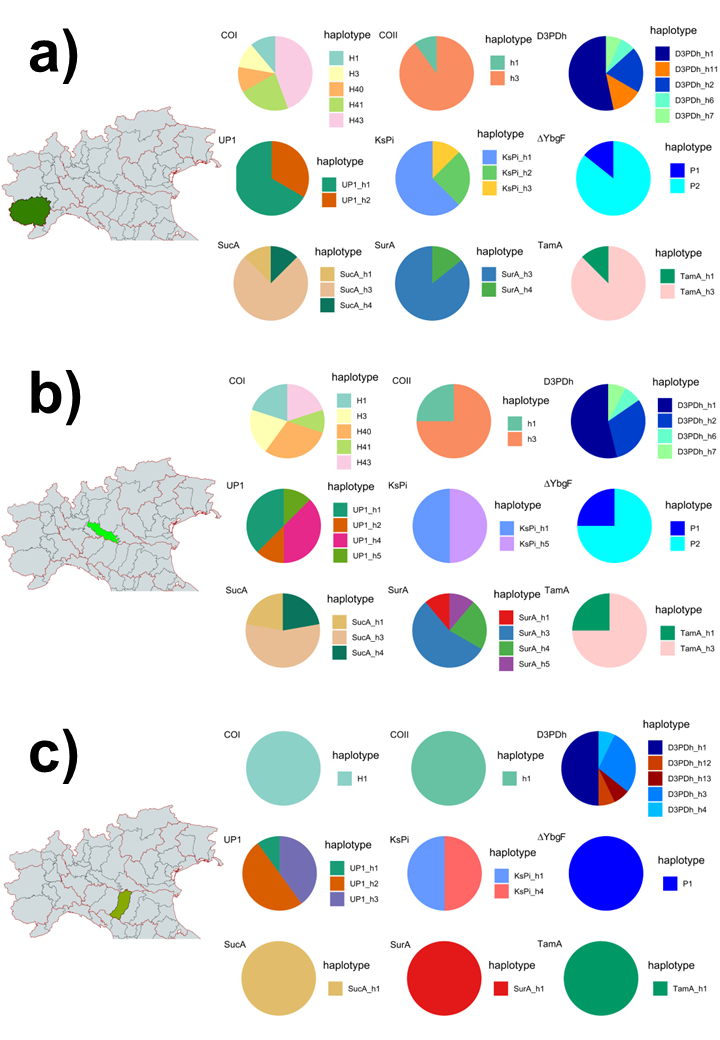


**Figure S2.** Haplotypes found for each marker under analysis in Italian populations. **A** Cuneo population **B** Cremona population **C** Reggio Emilia population. COI – COII = *H. halys* mitochondrial markers. D3PDh – UP1 – KsPi = *H. halys* nuclear markers. ΔYbgF – SucA – SurA – TamA = *P. carbekii* markers.


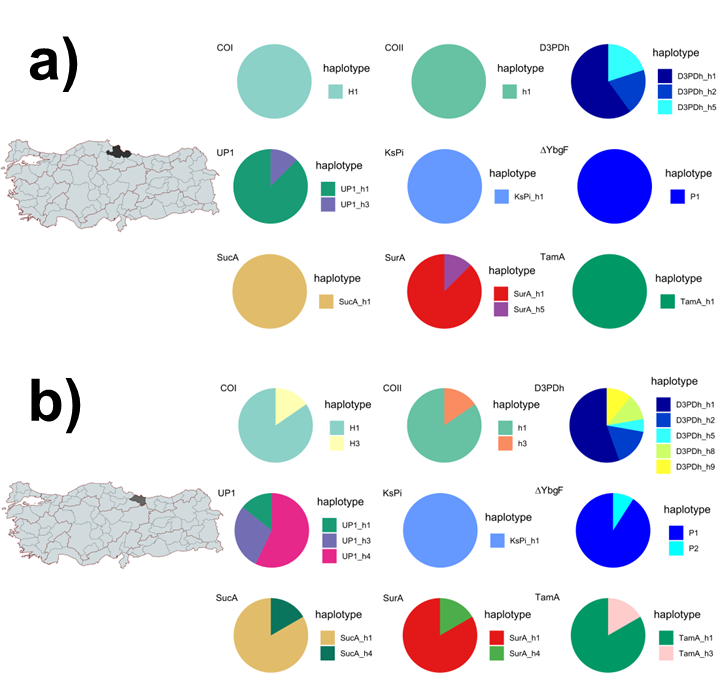


**Figure S3.** Haplotypes found for each marker under analysis in Turkish populations. **A** Samsun population **B** Ordu population. COI – COII = *H. halys* mitochondrial markers. D3PDh – UP1 – KsPi = *H. halys* nuclear markers. ΔYbgF – SucA – SurA – TamA = *P. carbekii* markers.


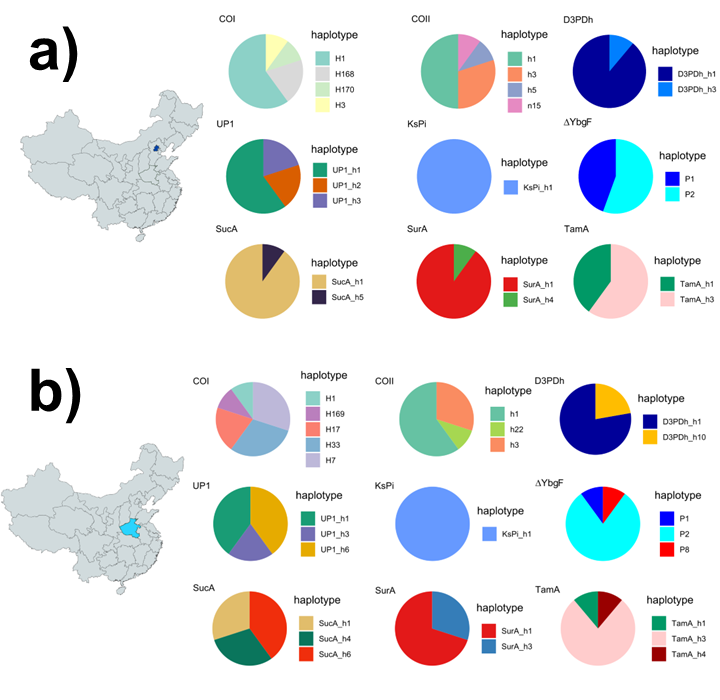


**Figure S4.** Haplotypes found for each marker under analysis in Chinese populations. **A** Beijing population **B** Henan population. COI – COII = *H. halys* mitochondrial markers. D3PDh – UP1 – KsPi = *H. halys* nuclear markers. ΔYbgF – SucA – SurA – TamA = *P. carbekii* markers.


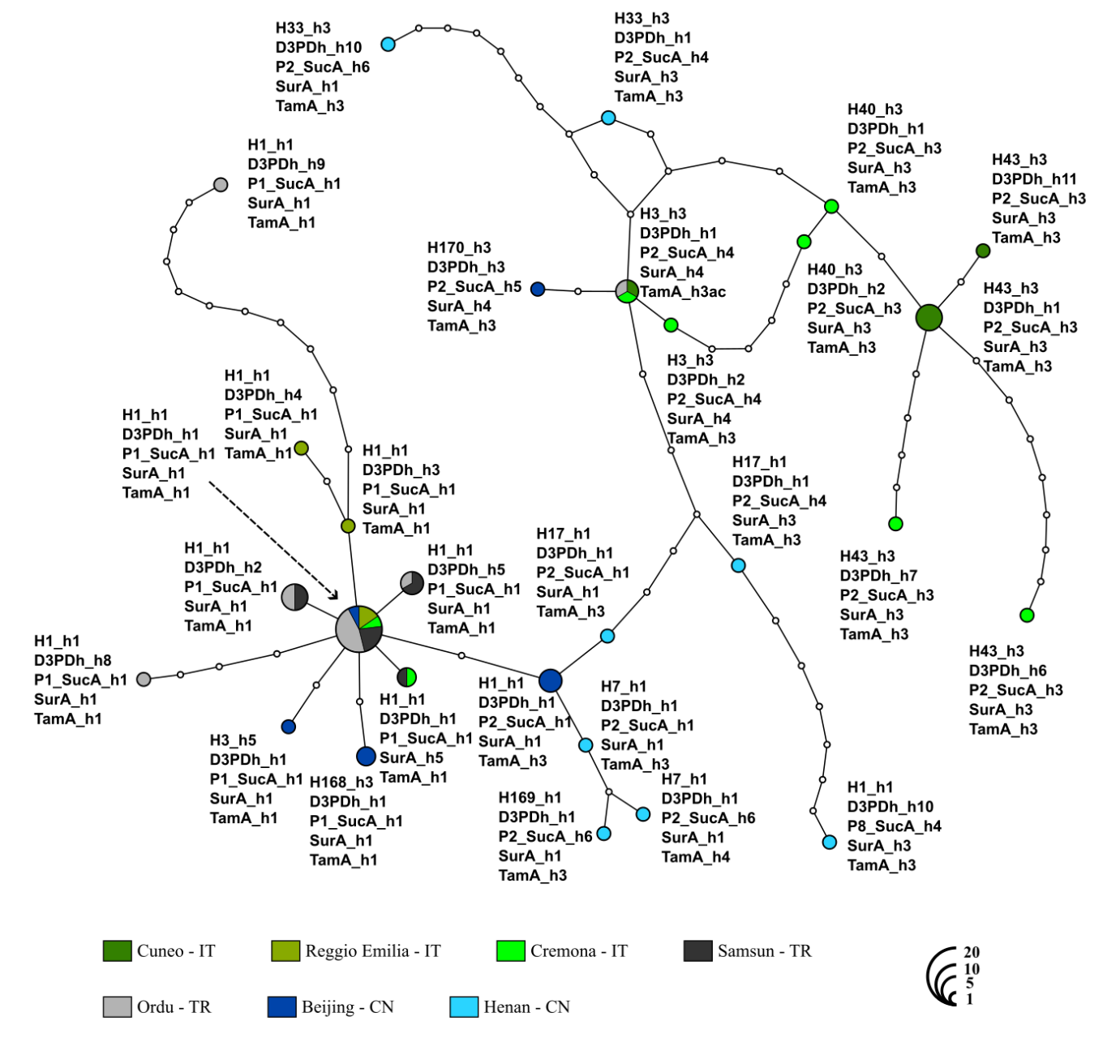


**Figure S5.** Haplotype distribution obtained combining the most informative markers from *Halyomorpha halys* and ‘*Candidatus* Pantoea carbekii’ in the populations under analysis. Circle size increases with the number of samples belonging to each haplotype. Small white circles represent putative or missing haplotypes forecasted by the analysis. Insect markers used: COI, COII and Hh_D3PDh. Symbiont markers used: ΔybgF, Pc_TamA, Pc_SucA and Pc_SurA.


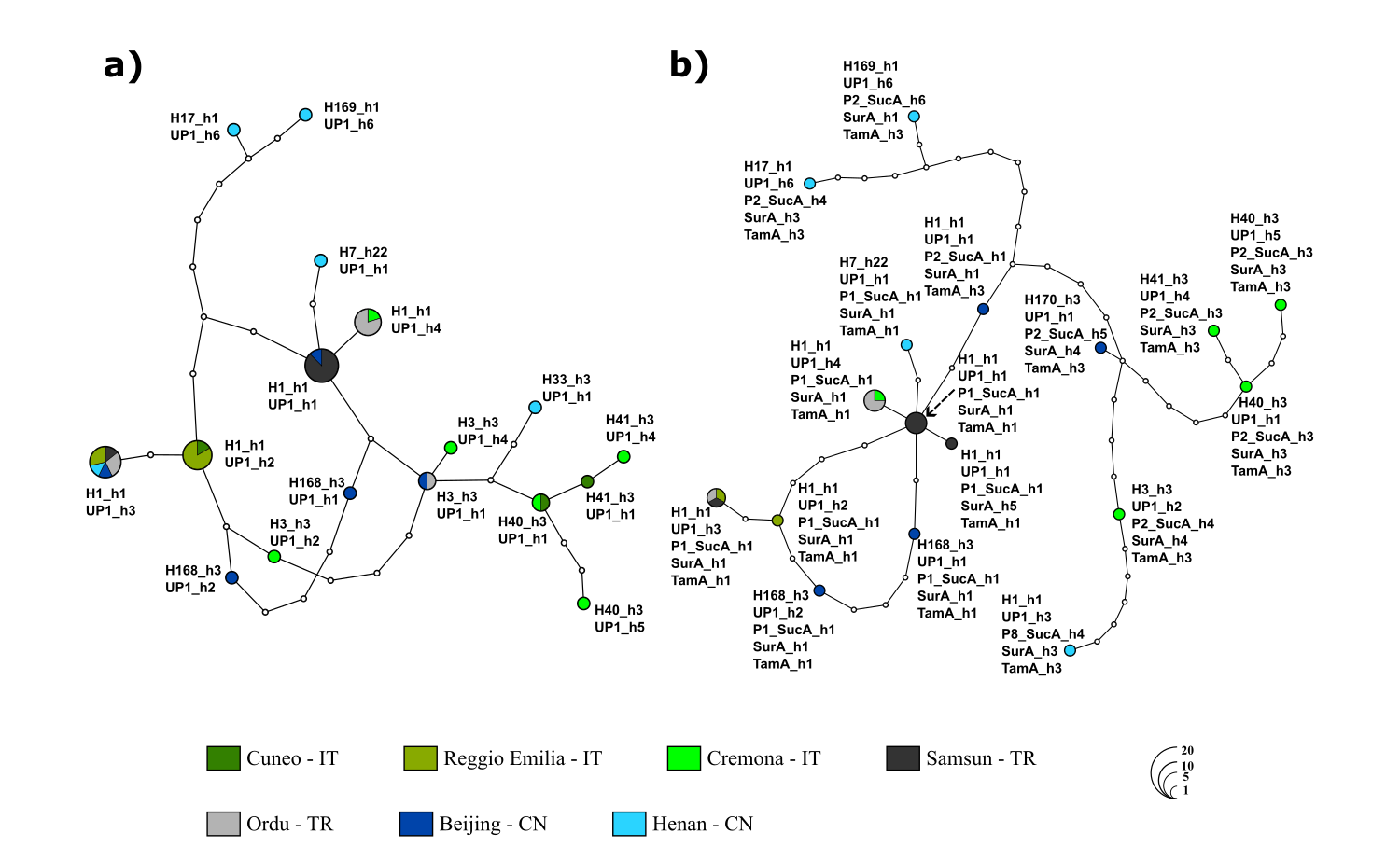
**Figure S6.** Haplotype distribution obtained using different MLST approaches. Circle size increases with the number of samples belonging to each haplotype. Small white circles represent putative or missing haplotypes forecasted by the analysis. **A** *Halyomorpha halys* haplotyping using COI + COII + Hh_UP1 markers **B** insect + symbiont haplotyping using *H. halys* markers COI + COII + Hh_UP1 and *‘Candidatus* Pantoea carbekii’ markers ΔybgF + Pc_TamA + Pc_SucA + Pc_SurA.


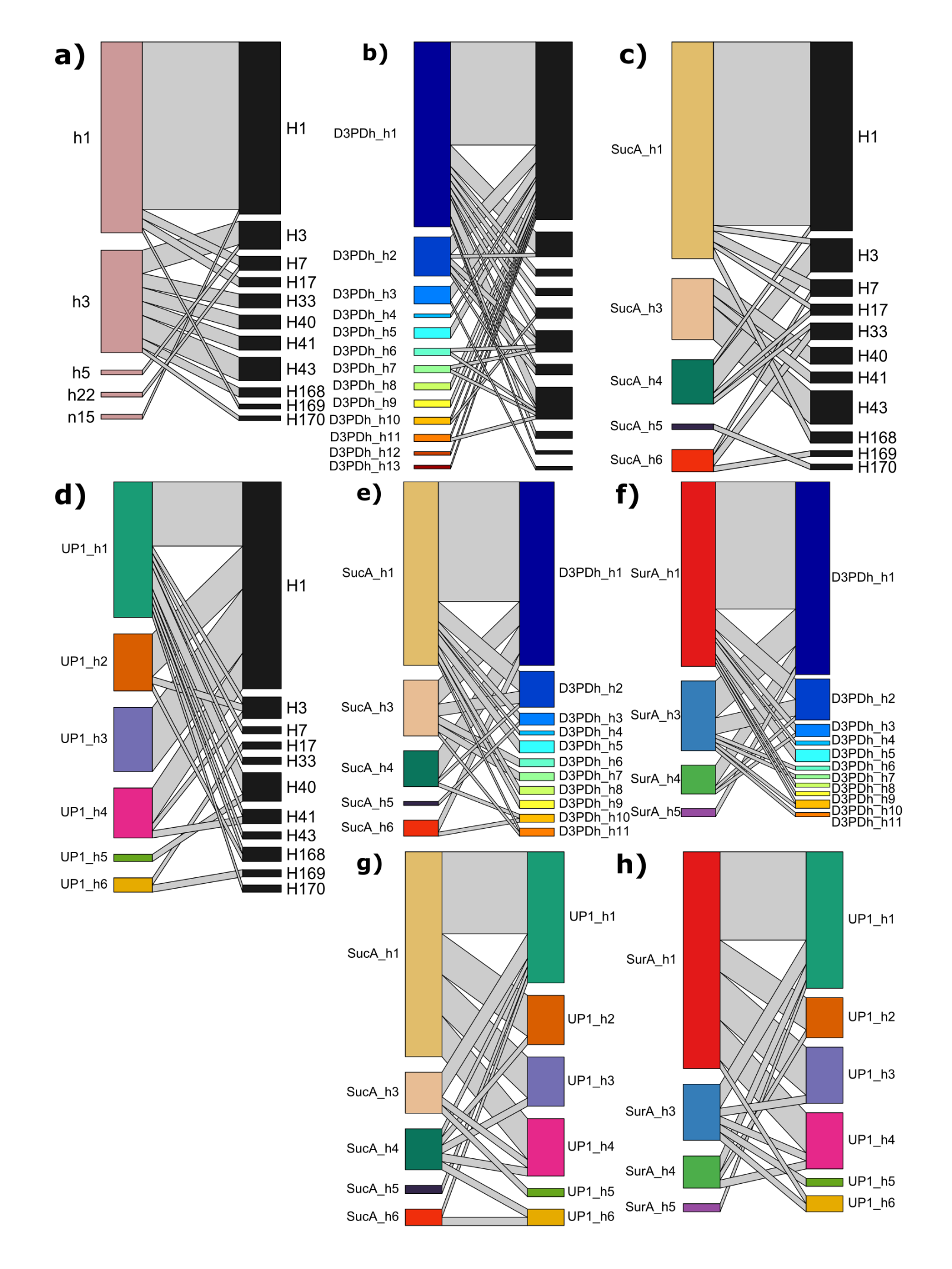


**Figure S7.** Bipartite interaction matrices between *Halyomorpha halys* and *‘Candidatus* Pantoea carbekii’ haplotypes for all the populations under analysis. The width of the conjunctions represents the frequency with which two haplotypes were found in a same individual. Different colours of the rectangles represent different haplotypes of the used marker. **A** *H. halys* COI with COII **B** *H. halys* COI with Hh_D3PDh **C** *H. halys* COI with *P. carbekii* Pc_SucA **D** *H. halys* COI with UP1 **E** *H. halys* Hh_D3PDh with *P. carbekii* Pc_SucA **F** *H. halys* Hh_D3PDh with *P. carbekii* Pc_SurA **G** *H. halys* Hh_UP1 with *P. carbekii* Pc_SucA **H** *H. halys* Hh_UP1 with *P. carbekii* Pc_SurA.


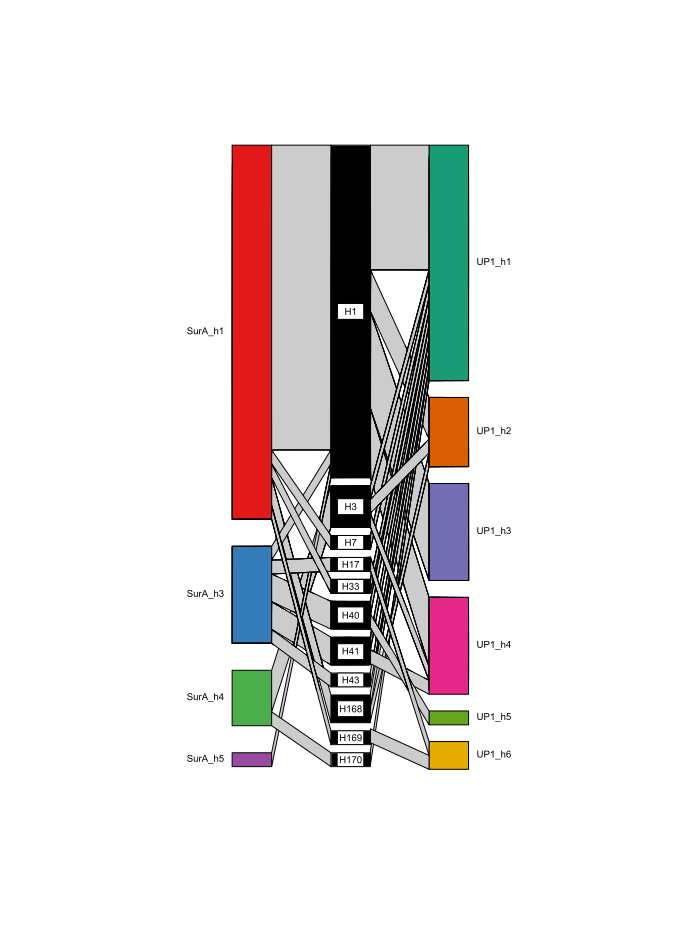


**Figure S8.** Bipartite networks of interaction between haplotypes of the *‘Candidatus* Pantoea carbekii’ Pc_SurA marker and *Halyomorpha halys* COI marker (left side), and between *H. halys* COI and Hh_UP1 markers (right side). The width of the conjunctions represents the frequency with which two haplotypes were found in a same holobiont. Different colours of the rectangles represent different haplotypes of the used marker.
