# Supplementary figures and images for: Multilocus sequence typing of the invasive pest Halyomorpha halys (Hemiptera: Pentatomidae) and associated endosymbiont reveals unexplored diversity
Source: Insect Sci. 2025 Apr 9;33(3):1187–205. doi: 10.1111/1744-7917.70034 (PMC13252635; doi:10.1111/1744-7917.70034)

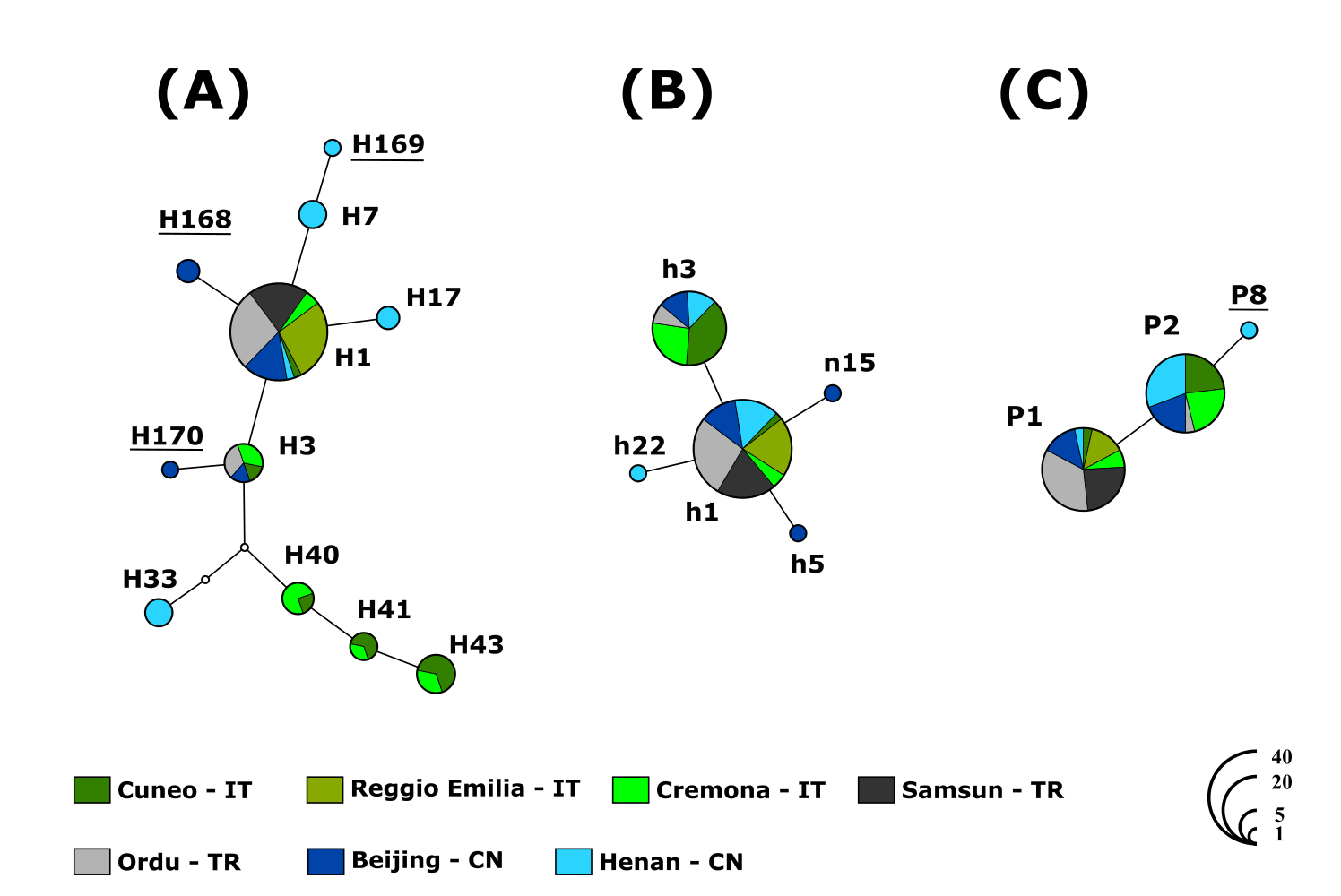

Supplement: Supplementary file 3 — Fig. S1 Haplotype distribution according to H. halys mitochondrial markers and “Ca. Pantoea carbekii” ΔybgF along the sampled populations. [file INS-33-1187-s006.png]

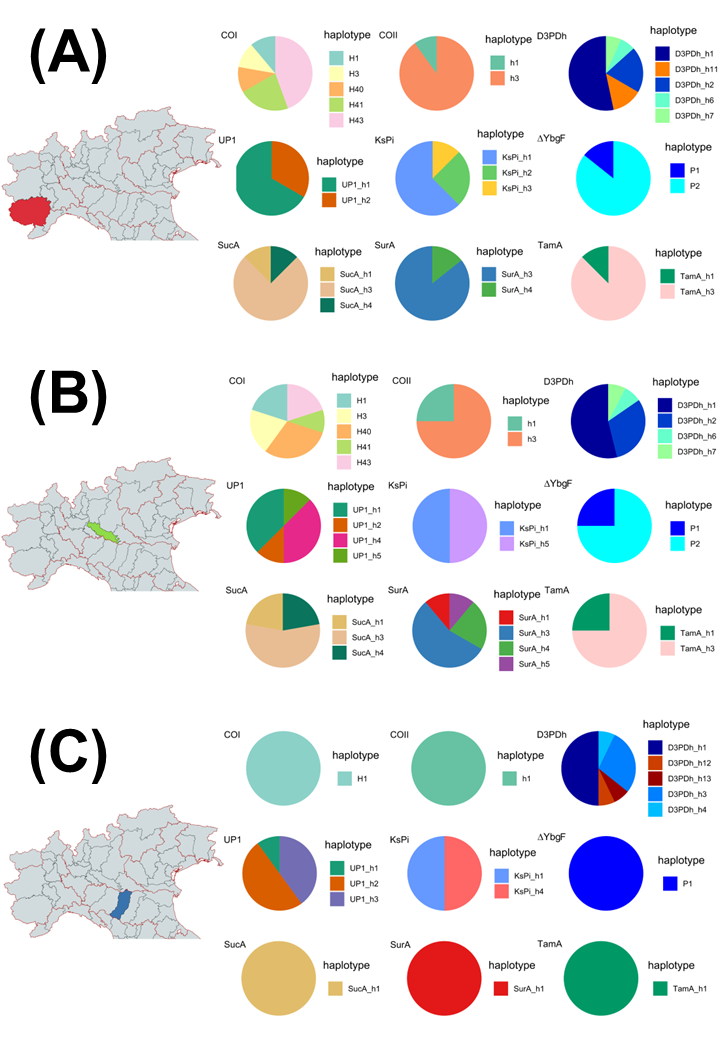

Supplement: Supplementary file 4 — Fig. S2 Haplotypes found for each marker under analysis in Italian populations. [file INS-33-1187-s009.PNG]

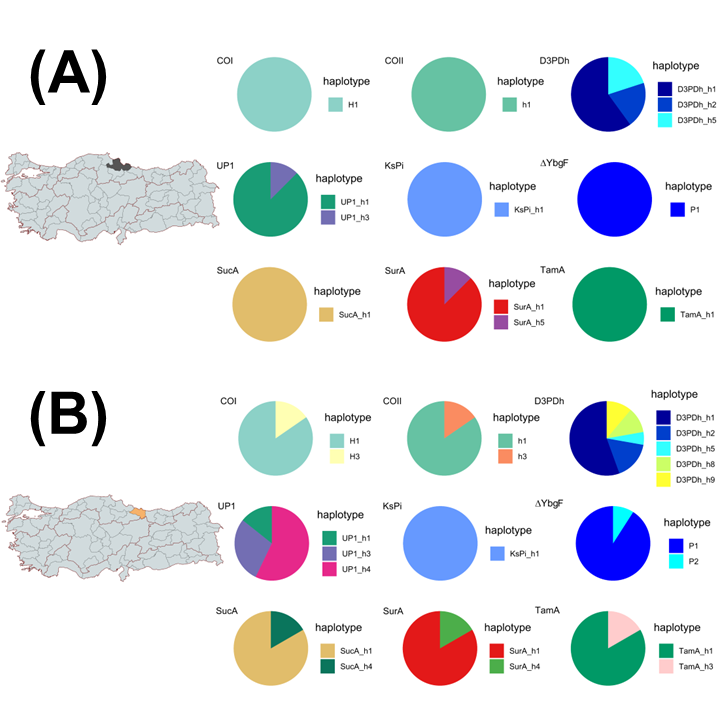

Supplement: Supplementary file 5 — Fig. S3 Haplotypes found for each marker under analysis in Turkish populations. [file INS-33-1187-s007.png]

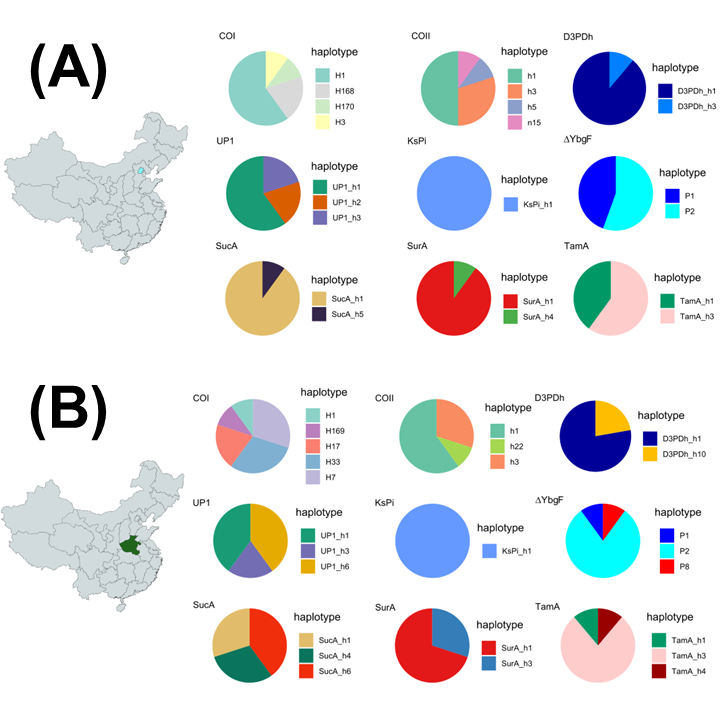

Supplement: Supplementary file 6 — Fig. S4 Haplotypes found for each marker under analysis in Chinese populations. [file INS-33-1187-s010.png]

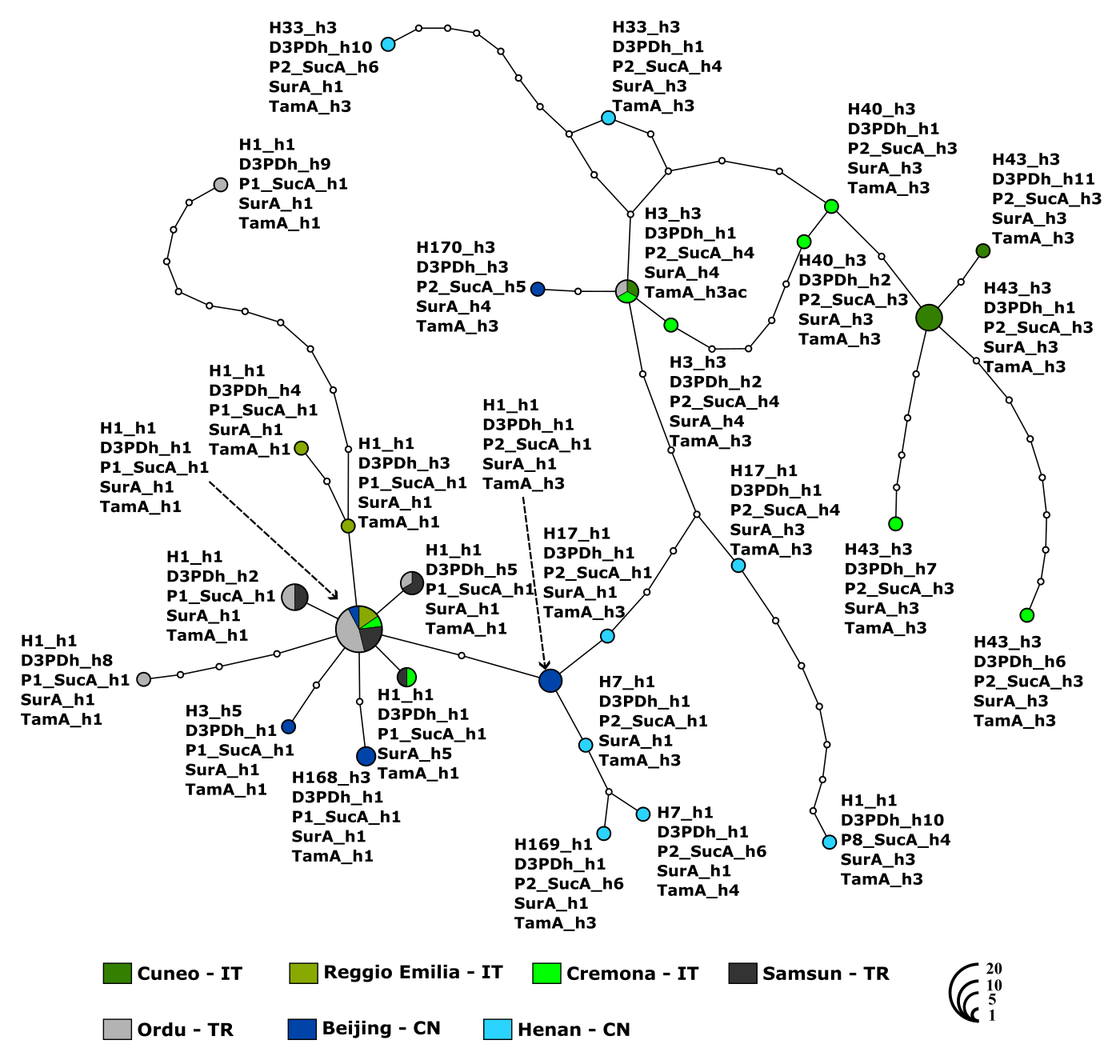

Supplement: Supplementary file 7 — Fig. S5 Haplotype distribution obtained combining the most informative markers from Halyomorpha halys and “Candidatus Pantoea carbekii” in the populations under analysis. [file INS-33-1187-s003.png]

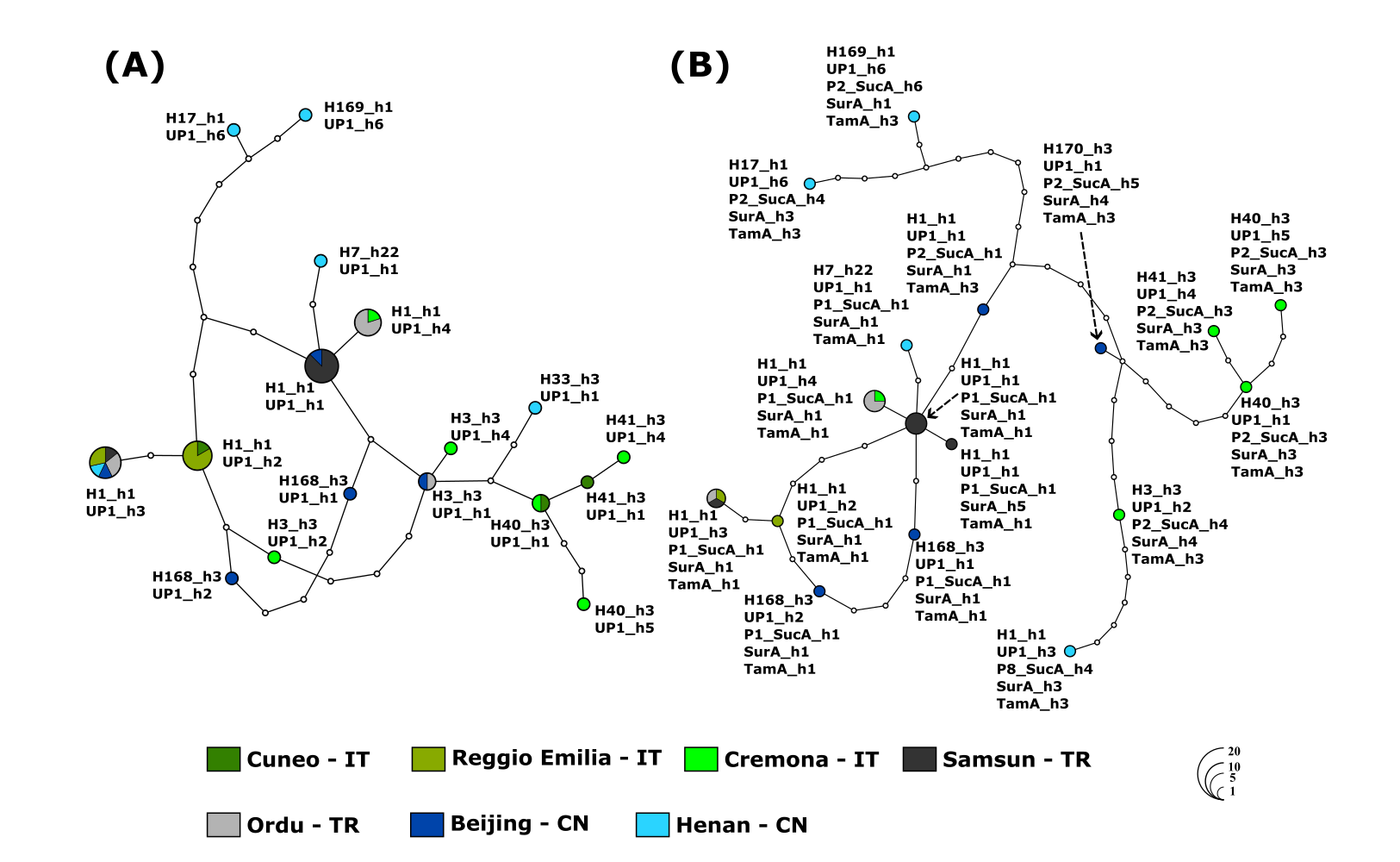

Supplement: Supplementary file 8 — Fig. S6 Haplotype distribution obtained using different MLST approaches. [file INS-33-1187-s005.png]

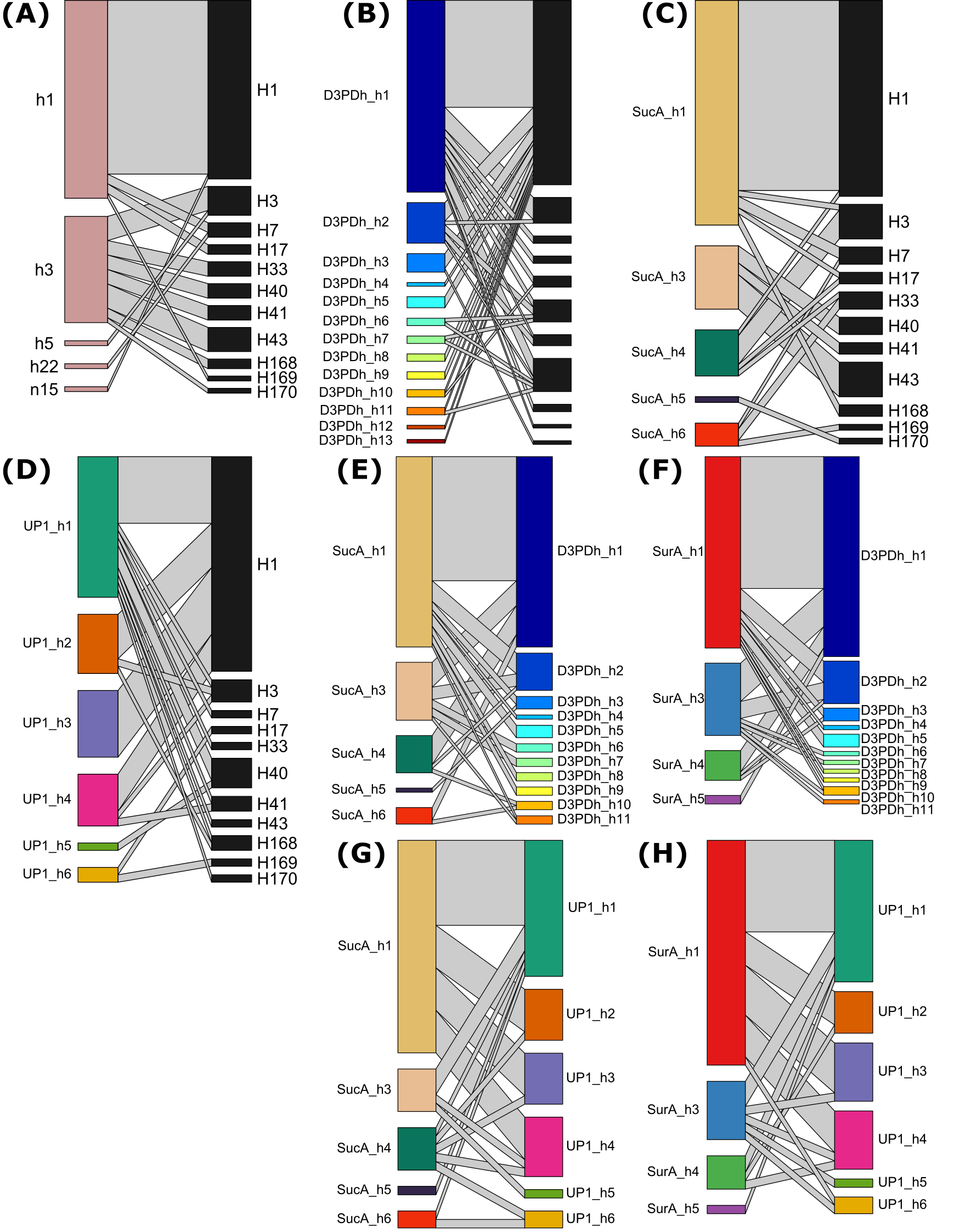

Supplement: Supplementary file 9 — Fig. S7 Bipartite interaction matrices between Halyomorpha halys and “Candidatus Pantoea carbekii” haplotypes for all the populations under analysis. [file INS-33-1187-s004.png]

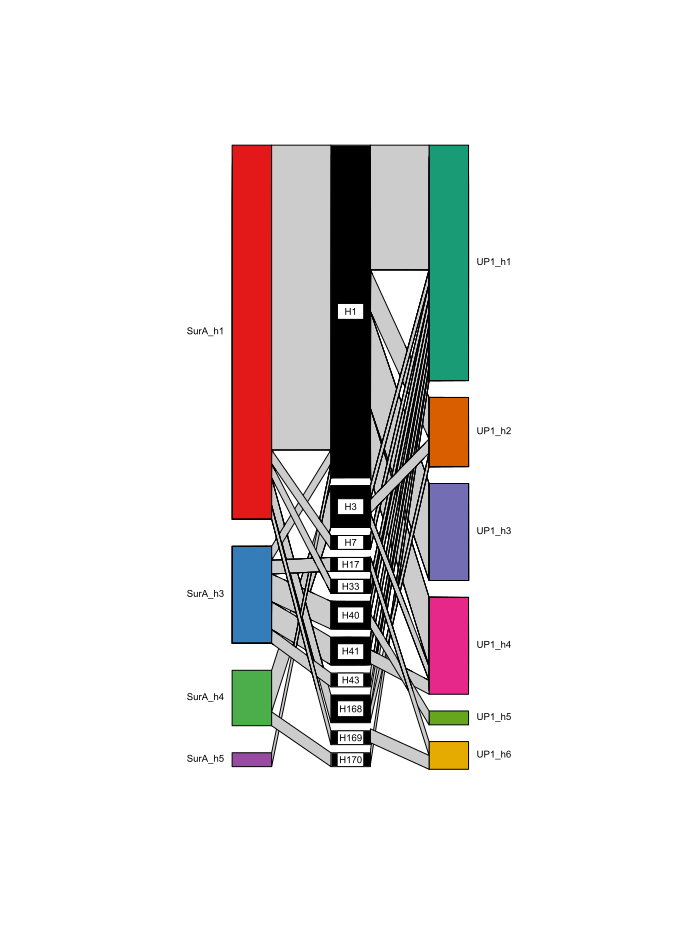

Supplement: Supplementary file 10 — Fig. S8 Bipartite networks of interaction between haplotypes of the “Candidatus Pantoea carbekii” Pc_SurA marker and Halyomorpha halys COI marker (left side), and between H. halys COI and Hh_UP1 markers (right side). [file INS-33-1187-s001.png]
